# Supplementary material for: Synthesis and Evaluation of Chloride-Substituted Ramalin Derivatives for Alzheimer’s Disease Treatment
Source: Molecules. 2024 Aug 5;29(15):3701. doi: 10.3390/molecules29153701 (PMC11313798; doi:10.3390/molecules29153701)
Supplement: Supplementary file 1 [file molecules-29-03701-s001.zip › molecules-3122513-supplementary.pdf]

molecules

Supporting information

Synthesis and Evaluation of Chloride-Substituted Ramalin Derivatives for Alzheimer's Disease Treatment

Tai Kyoung Kim [1,†], Yongeun Cho [2,†], Jaewon Kim [1,3], Jeongmi Lee [2], Ju-Mi Hong [1], Heewon Cho [2], Jun-Sik Kim [2], Yeongyeong Lee [2], Kyung Hee Kim [1,4], Il-Chan Kim [1], Se Jong Han [1], Hyuncheol Oh [5], Dong-Gyu Jo [2,*] and Joung Han Yim [1,*]

[1] Division of Polar Life Sciences, Korea Polar Research Institute, Incheon 21990, Republic of Korea; tkkim@kopri.re.kr (T.K.K.); ashcercle@kopri.re.kr (J.K.); wnal5555@kopri.re.kr (J.-M.H.); kh313@kopri.re.kr (K.H.K.); ickim@kopri.re.kr (I.-C.K.); hansj@kopri.re.kr (S.J.H.)
[2] School of Pharmacy, Sungkyunkwan University, Suwon 16419, Republic of Korea; okcho9307@naver.com (Y.C.); jungmileedy@naver.com (J.L.); hwcho1012@gmail.com (H.C.); khws123486@nate.com (J.-S.K.); dusrud1129@nate.com (Y.L.)
[3] Department of Plant Biotechnology, Korea University, Seoul 02841, Republic of Korea
[4] Department of Chemistry, Hanseo University, Seosan 31962, Republic of Korea
[5] College of Pharmacy, Wonkwang University, Iksan 54538, Republic of Korea; hoh@wcu.ac.kr
* Correspondence: jodg@skku.edu (D.-G.J.); jhyim@kopri.re.kr (J.H.Y.); Tel.: +82-31-290-7776 (D.-G.J.); +82-32-760-5540 (J.H.Y.); Fax: +82-32-760-5509 (J.H.Y.)
† These authors contributed equally to this work.

Context .... page

N[5]*-((2-chlorophenyl)amino)-L-glutamine* (**RA-2Cl**)

Figure S1. ^{1}H NMR spectrum of **RA-2Cl** 4

Figure S2. ^{13}C NMR spectrum of **RA-2Cl** 4

Figure S3. DEPT spectrum of **RA-2Cl** 5

Figure S4. COSY spectrum of **RA-2Cl** 5

Figure S5. HSQC spectrum of **RA-2Cl** 6

Figure S6. HMBC spectrum of **RA-2Cl** 6

Figure S7. HRESIMS spectrum of **RA-2Cl** 7

N[5]*-((3-chlorophenyl)amino)-L-glutamine* (**RA-3Cl**)

Figure S8. ^{1}H NMR spectrum of **RA-3Cl** 7

Figure S9. ^{13}C NMR spectrum of **RA-3Cl** 8

Figure S10. DEPT spectrum of **RA-3Cl** 8

Figure S11. COSY spectrum of **RA-2Cl** 9

Figure S12. HSQC spectrum of **RA-2Cl** 9

Figure S13. HMBC spectrum of **RA-2Cl** 10

Figure S14. HRESIMS spectrum of **RA-2Cl** 10

N[5]*-((4-chlorophenyl)amino)-L-glutamine* (**RA-4Cl**)

Figure S15. ^{1}H NMR spectrum of **RA-4Cl** 11

Figure S16. ^{13}C NMR spectrum of **RA-4Cl** 11

Figure S17. DEPT spectrum of **RA-4Cl** 12

Figure S18. HSQC spectrum of **RA-4Cl** 12

Figure S19. HMBC spectrum of **RA-4Cl** 13

Figure S20. HRESIMS spectrum of **RA-4Cl** 13

N[5]*-((2,3-dichlorophenyl)amino)-L-glutamine* (**RA-23Cl**)

Figure S21. ^{1}H NMR spectrum of **RA-23Cl** 14

Figure S22. ^{13}C NMR spectrum of **RA-23Cl** 14

Figure S23. DEPT spectrum of **RA-23Cl** 15

Figure S24. COSY spectrum of **RA-23Cl** 15

Figure S25. HSQC spectrum of **RA-23Cl** 16

Figure S26. HRESIMS spectrum of **RA-23Cl** 16

N^5-((2,4-dichlorophenyl)amino)-L-glutamine (**RA-24Cl**)
Figure S27. ^{1}H NMR spectrum of **RA-24Cl** 17
Figure S28. ^{13}C NMR spectrum of **RA-24Cl**........ 17
Figure S29. DEPT spectrum of **RA-24Cl** 18
Figure S30. COSY spectrum of **RA-24Cl**........ 18
Figure S31. HSQC spectrum of **RA-24Cl** 19
Figure S32. HMBC spectrum of **RA-24Cl** 19
Figure S33. HRESIMS spectrum of **RA-24Cl**........ 20
N^5-((2,6-dichlorophenyl)amino)-L-glutamine (**RA-26Cl**)
Figure S34. ^{1}H NMR spectrum of **RA-26Cl** 20
Figure S35. ^{13}C NMR spectrum of **RA-26Cl** 21
Figure S36. DEPT spectrum of **RA-26Cl** 21
Figure S37. COSY spectrum of **RA-26Cl** 22
Figure S38. HSQC spectrum of **RA-26Cl** 22
Figure S39. HMBC spectrum of **RA-26Cl** 23
Figure S40. HRESIMS spectrum of **RA-26Cl**........ 23
N^5-((3,4-dichlorophenyl)amino)-L-glutamine (**RA-34Cl**)
Figure S41. ^{1}H NMR spectrum of **RA-34Cl** 24
Figure S42. ^{13}C NMR spectrum of **RA-34Cl**........ 24
Figure S43. DEPT spectrum of **RA-34Cl** 25
Figure S44. COSY spectrum of **RA-34Cl**........ 25
Figure S45. HSQC spectrum of **RA-34Cl** 26
Figure S46. HMBC spectrum of **RA-34Cl** 26
Figure S47. HRESIMS spectrum of **RA-34Cl**........ 27
N^5-((2,4-difluorophenyl)amino)-L-glutamine (**RA-35Cl**)
Figure S48. ^{1}H NMR spectrum of **RA-35Cl** 27
Figure S49. ^{13}C NMR spectrum of **RA-35Cl**........ 28
Figure S50. DEPT spectrum of **RA-35Cl** 28
Figure S51. COSY spectrum of **RA-35Cl**........ 29
Figure S52. HSQC spectrum of **RA-35Cl** 29
Figure S53. HMBC spectrum of **RA-35Cl** 30
Figure S54. HRESIMS spectrum of **RA-35Cl**........ 30

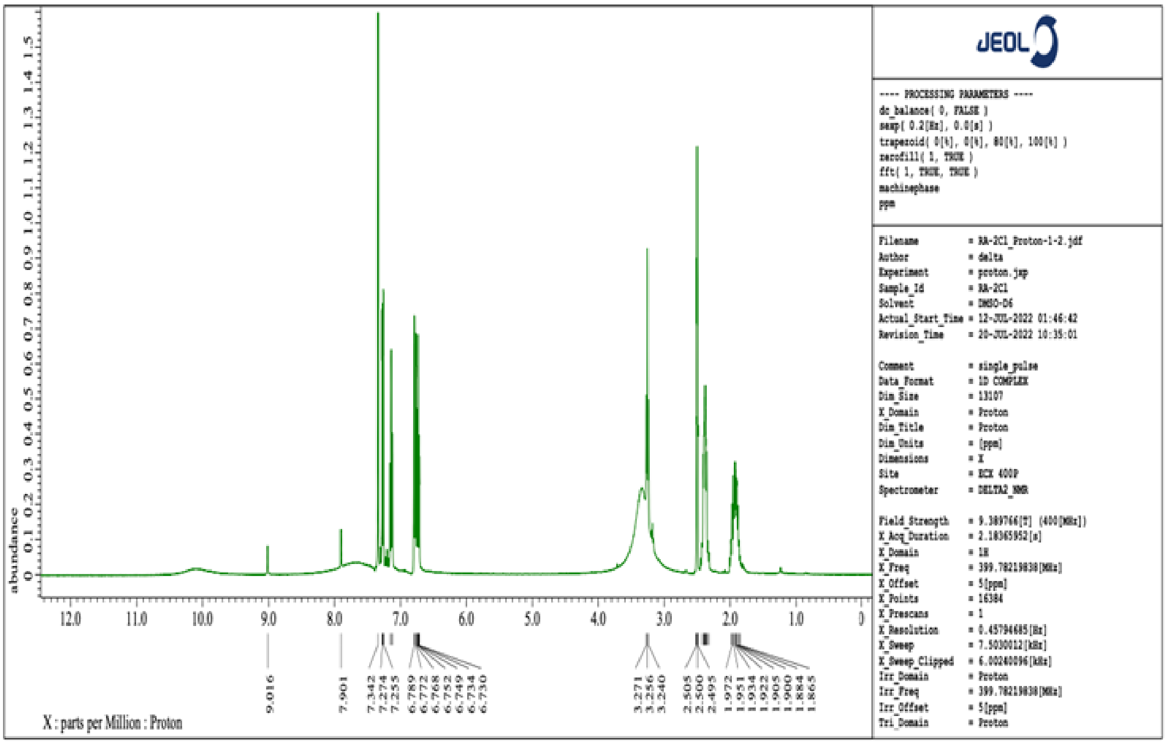

Figure S1: ^{1}H NMR (400 MHz) spectrum of **RA-2Cl**

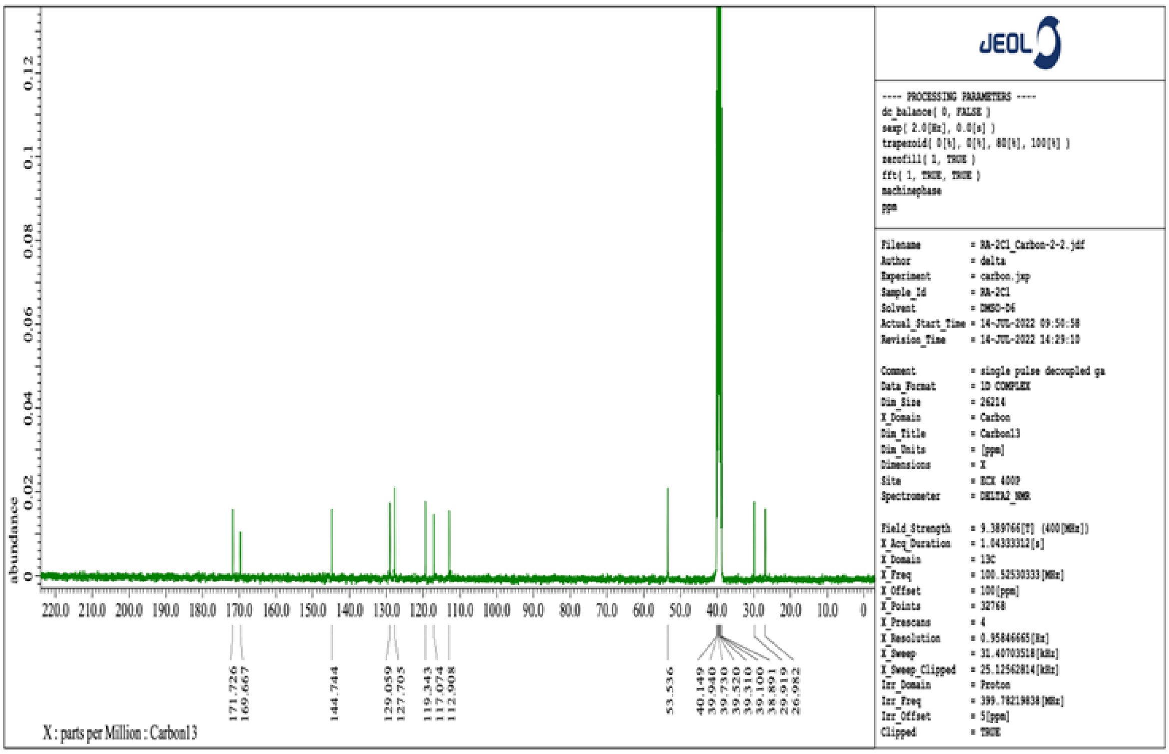

Figure S2: ^{13}C NMR (100 MHz) spectrum of **RA-2Cl**

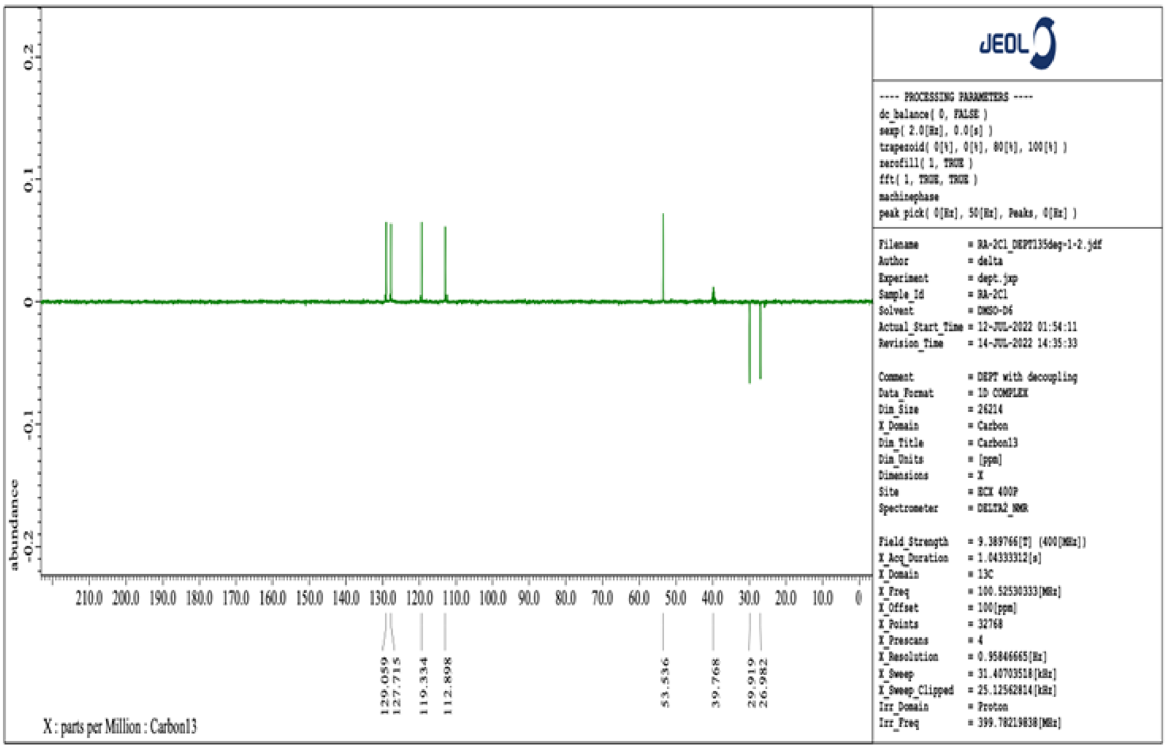

Figure S3: DEPT spectrum of **RA-2Cl**

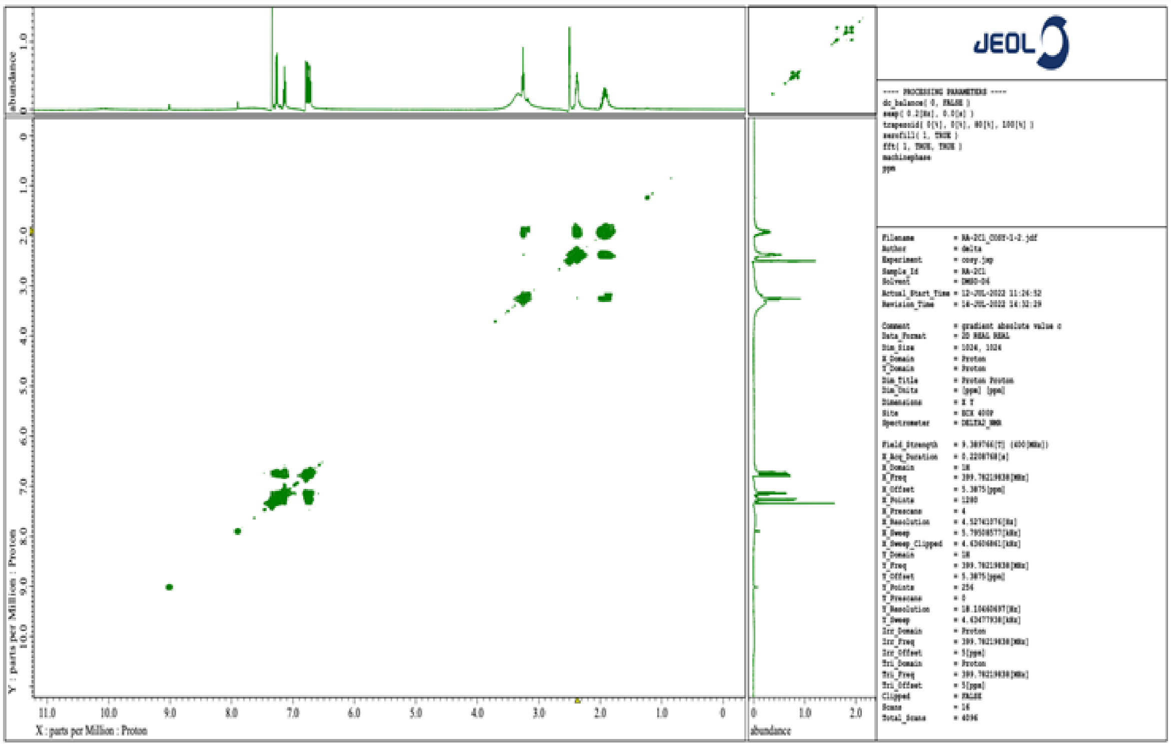

Figure S4: COSY spectrum of **RA-2Cl**

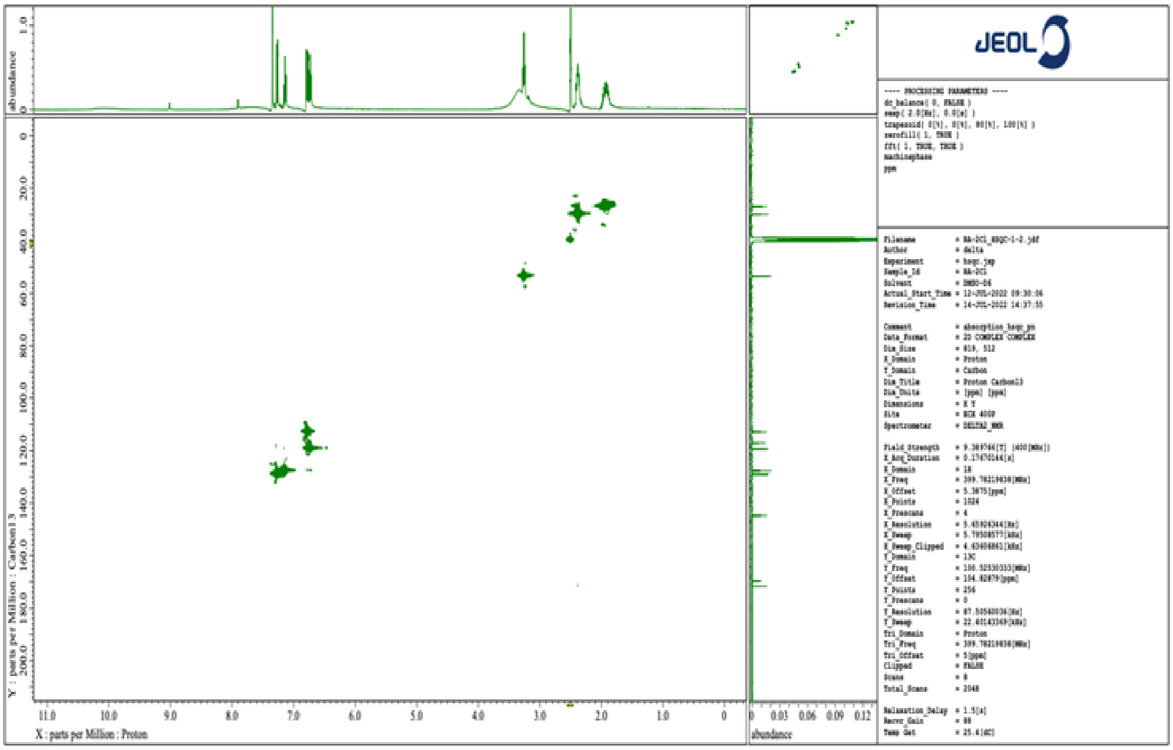

Figure S5: HMQC spectrum of **RA-2Cl**

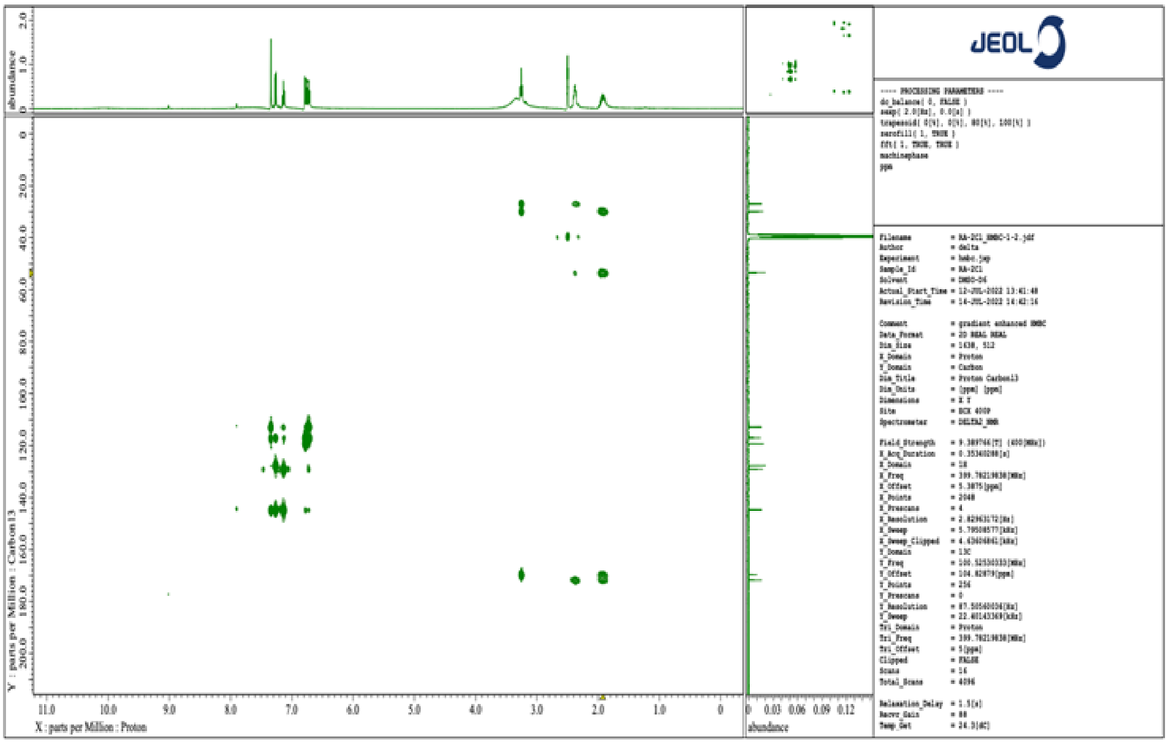

Figure S6: HMBC spectrum of **RA-2Cl**

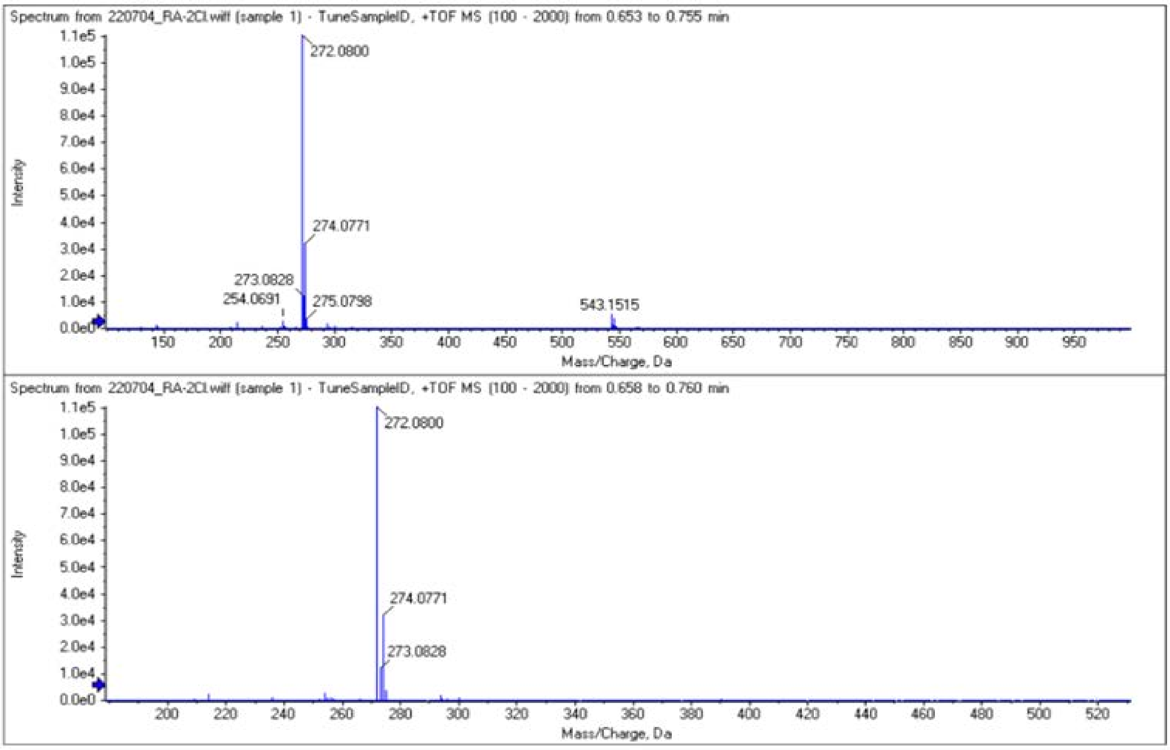

Figure S7: HRESIMS spectrum of **RA-2Cl**

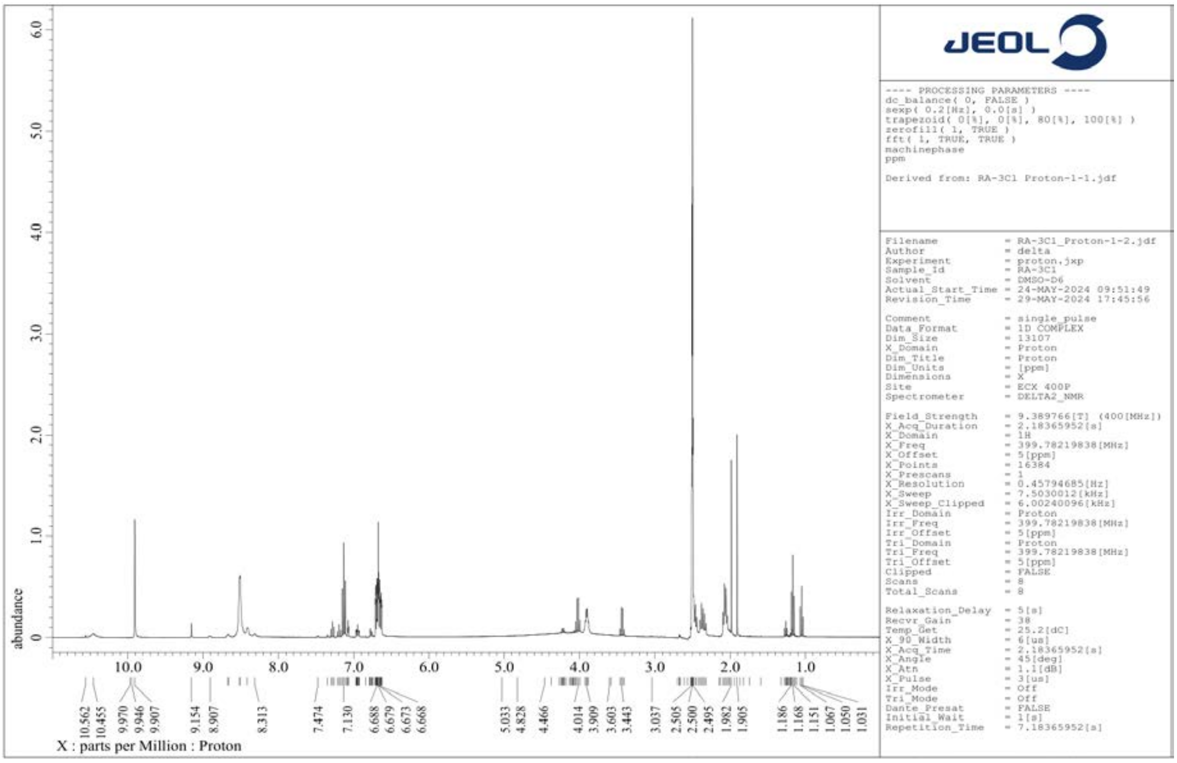

Figure S8: ^{1}H NMR (400 MHz) spectrum of **RA-3Cl**

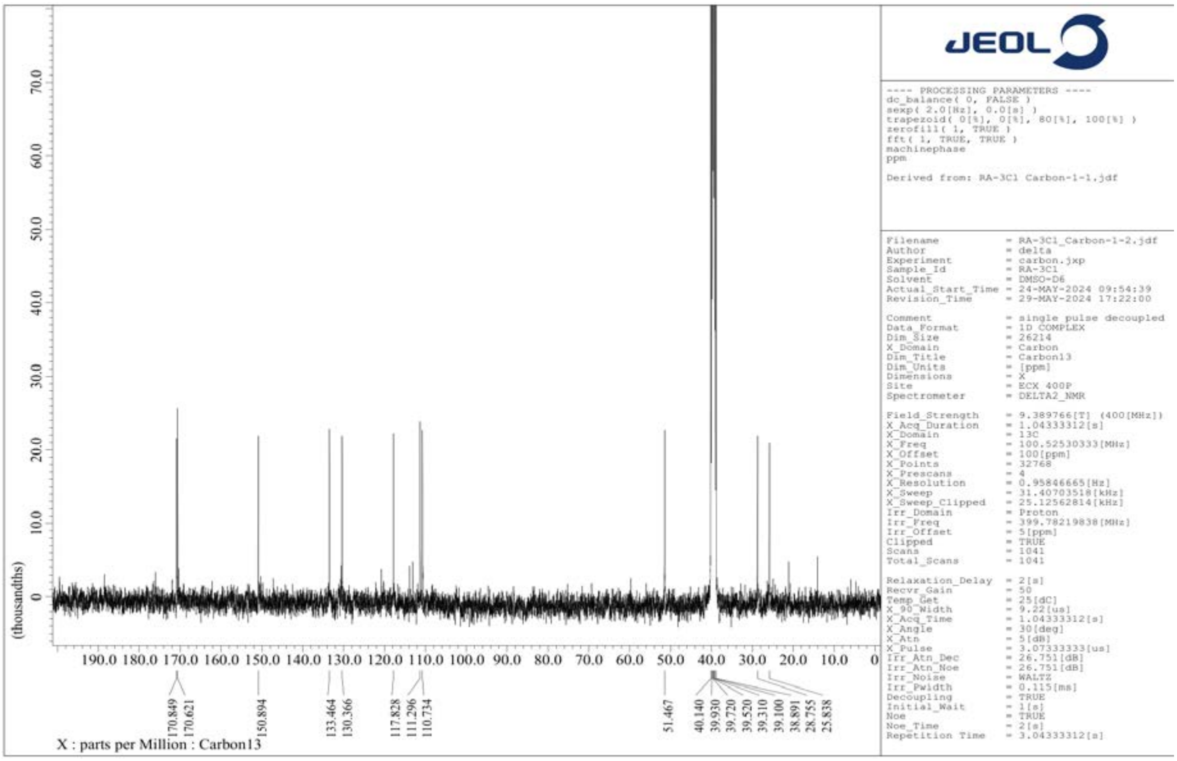

Figure S9: ^{13}C NMR (100 MHz) spectrum of **RA-3Cl**

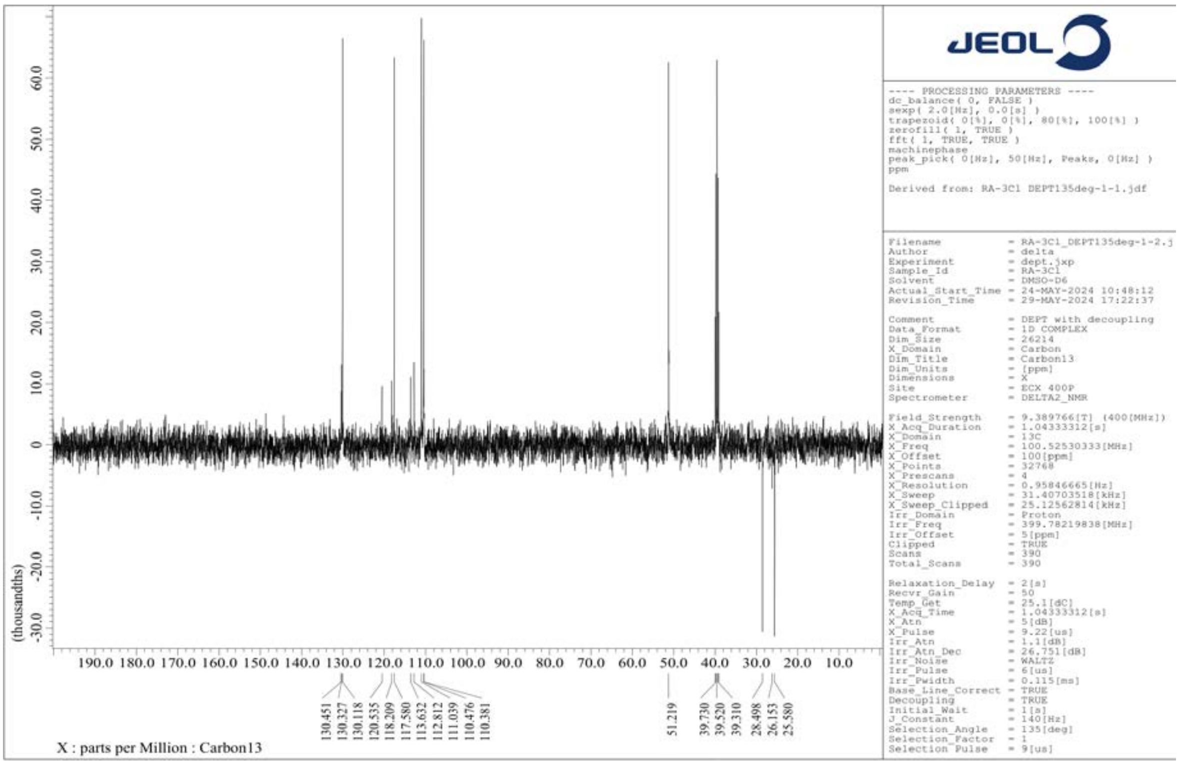

Figure S10: DEPT spectrum of **RA-3Cl**

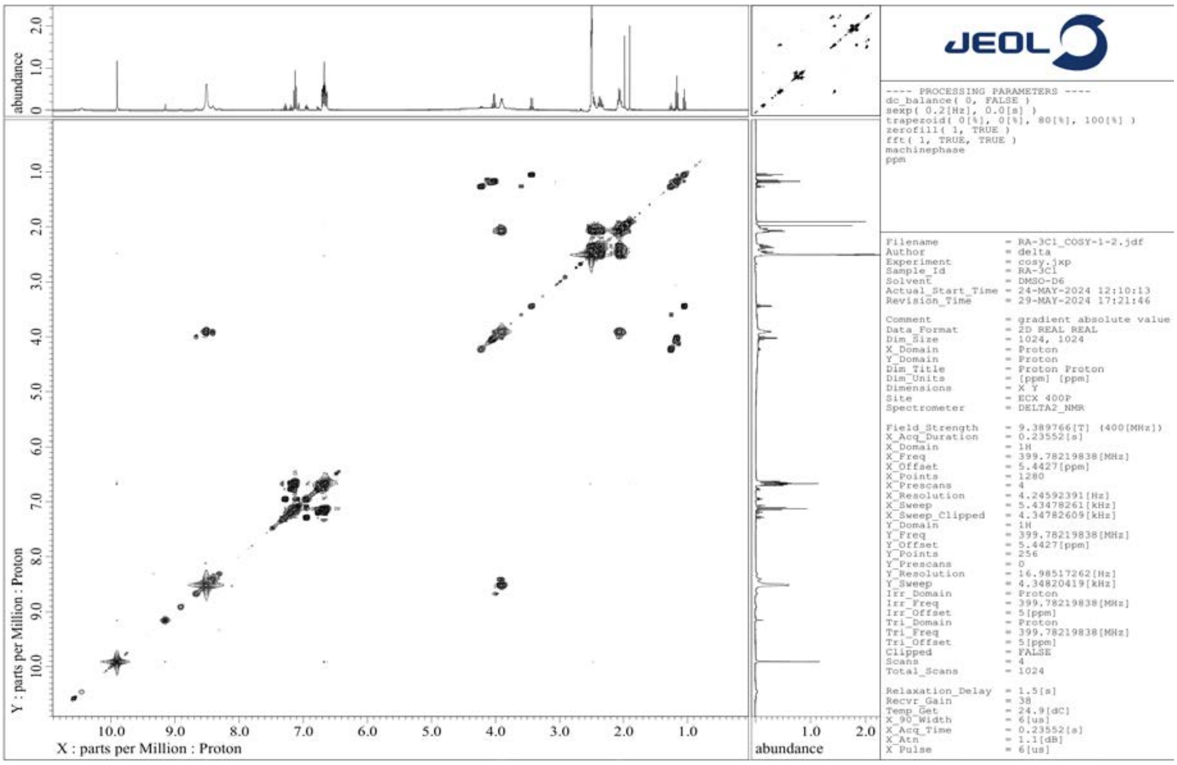

Figure S11: COSY spectrum of **RA-3Cl**

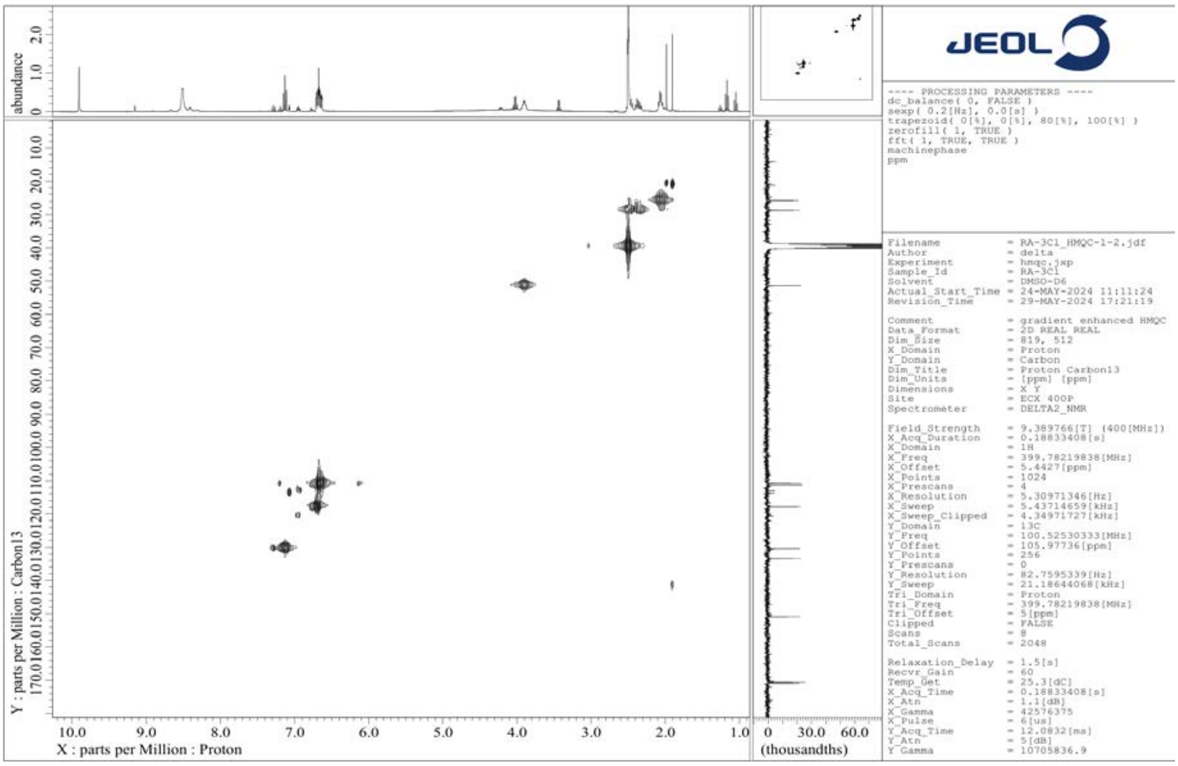

Figure S12: HMQC spectrum of **RA-3Cl**

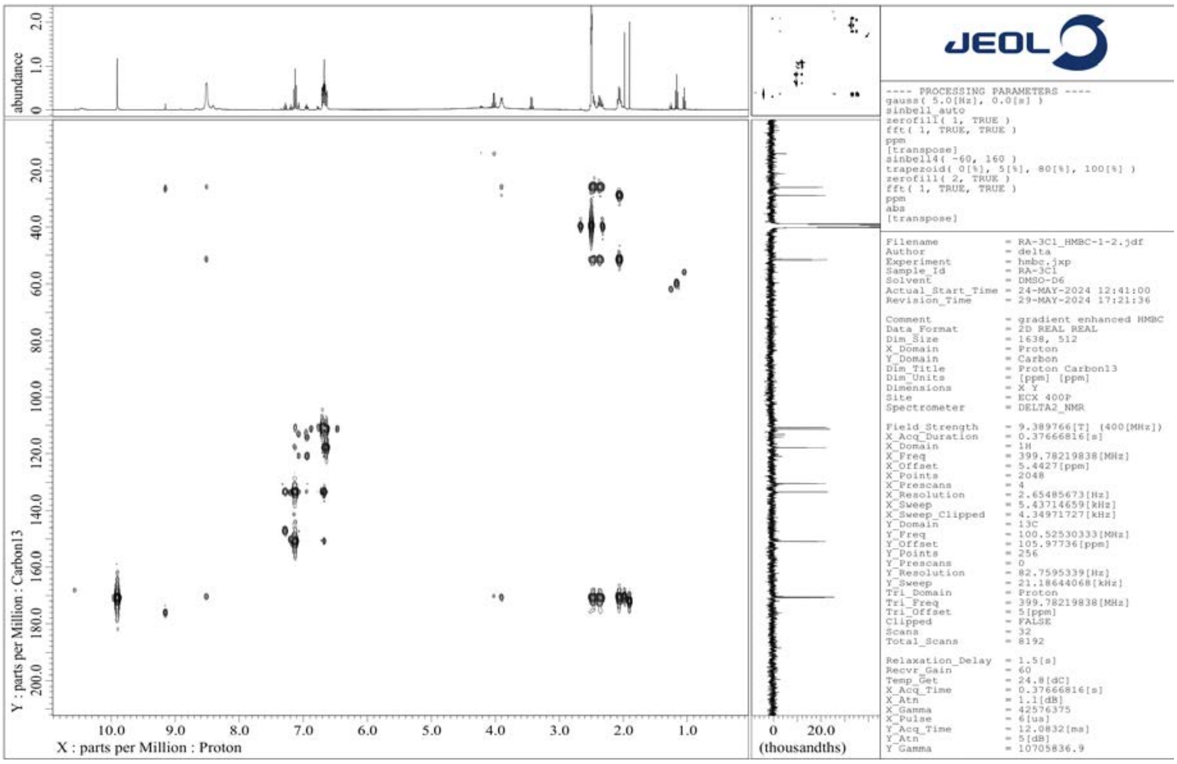

Figure S13: HMBC spectrum of **RA-3Cl**

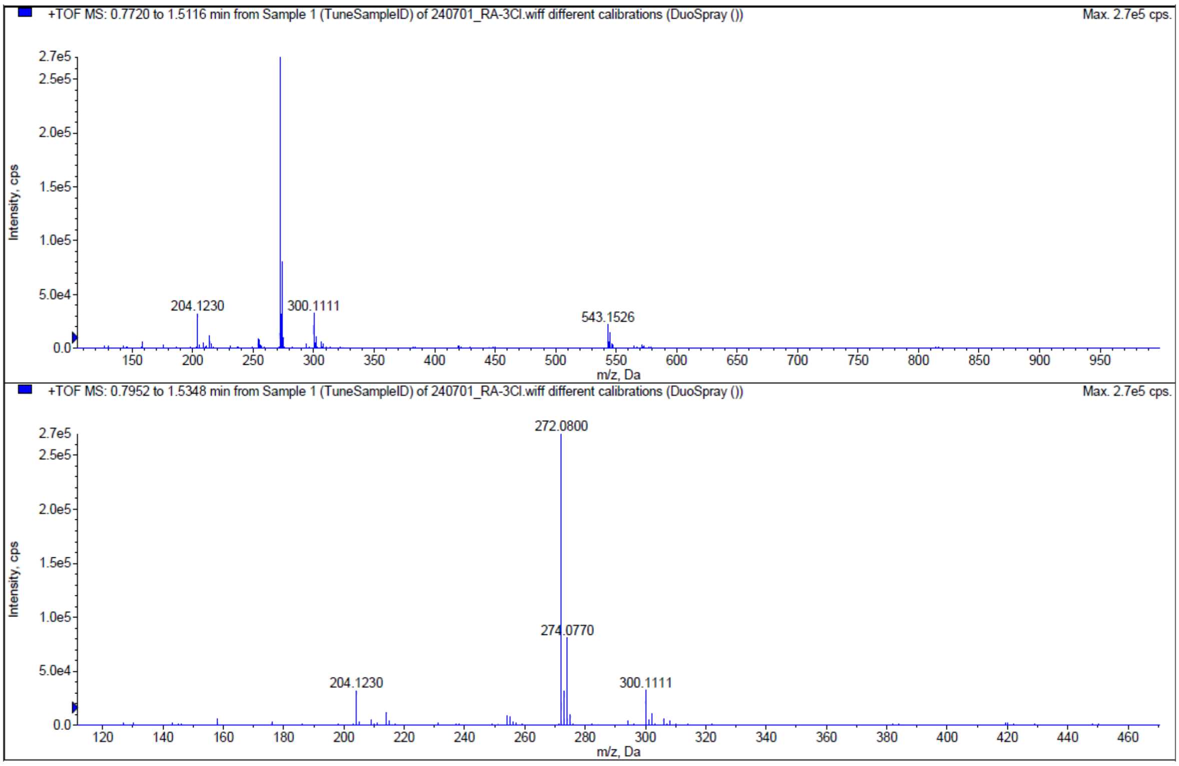

Figure S14: HRESIMS spectrum of **RA-3Cl**

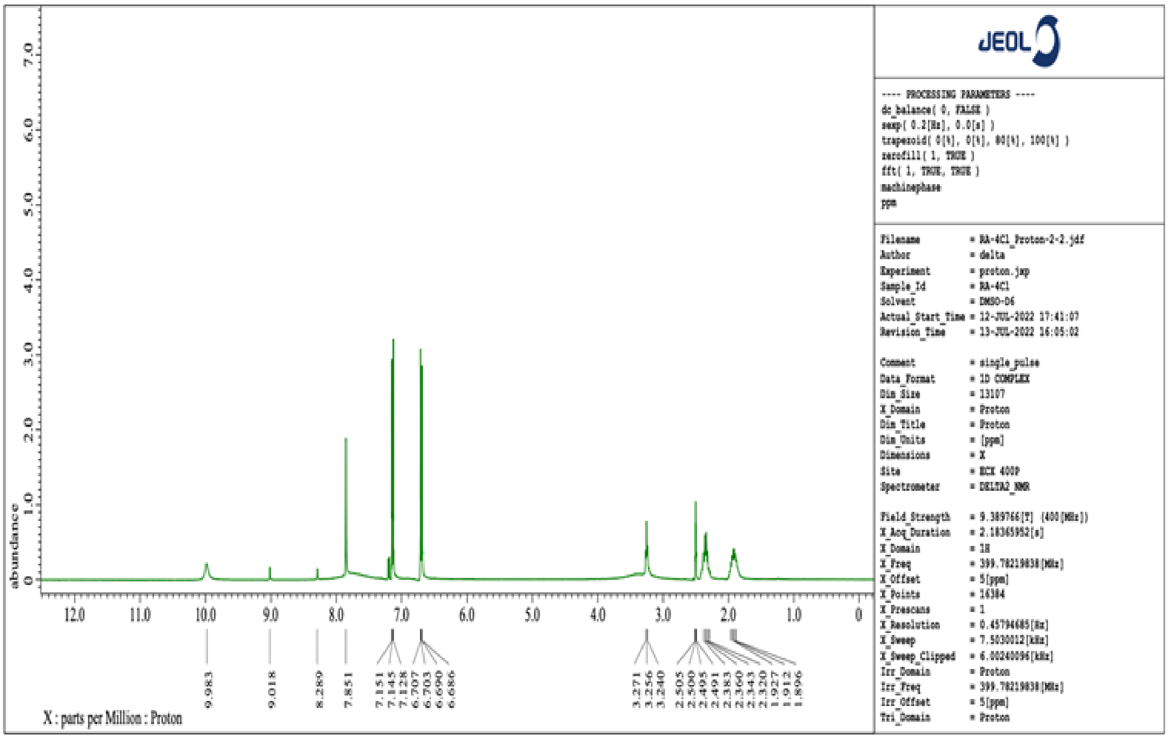

Figure S15: ^{1}H NMR (400 MHz) spectrum of **RA-4Cl**

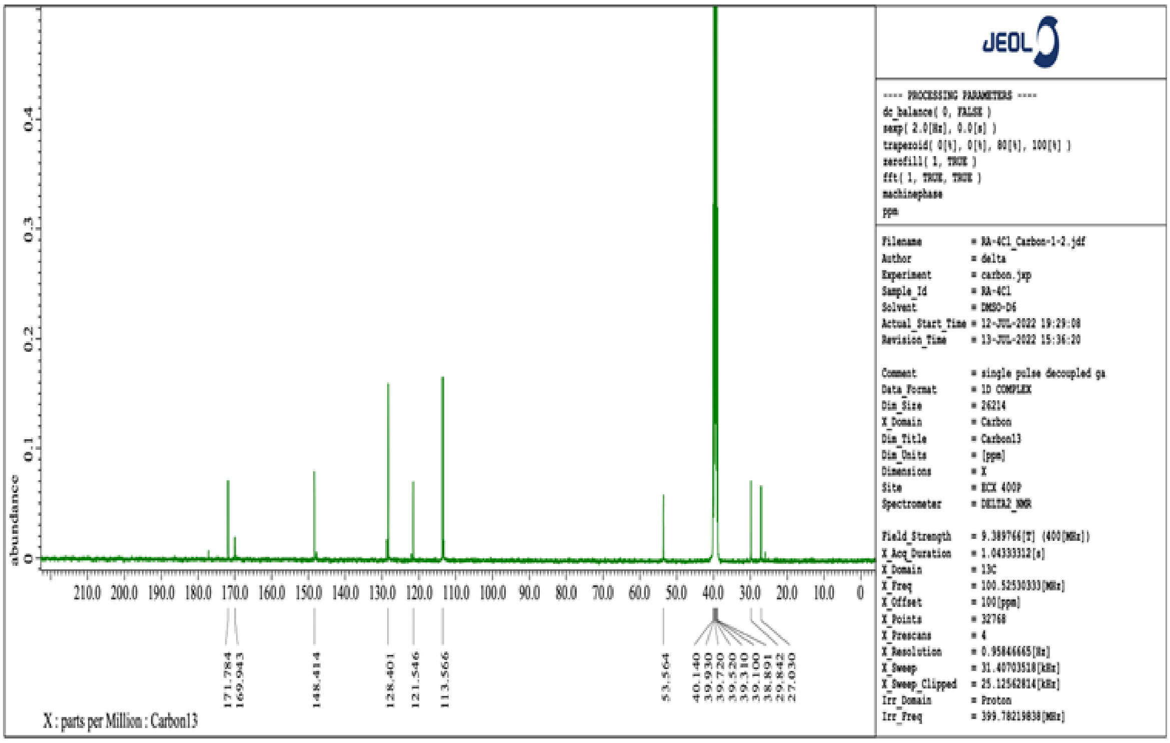

Figure S16: ^{13}C NMR (100 MHz) spectrum of **RA-4Cl**

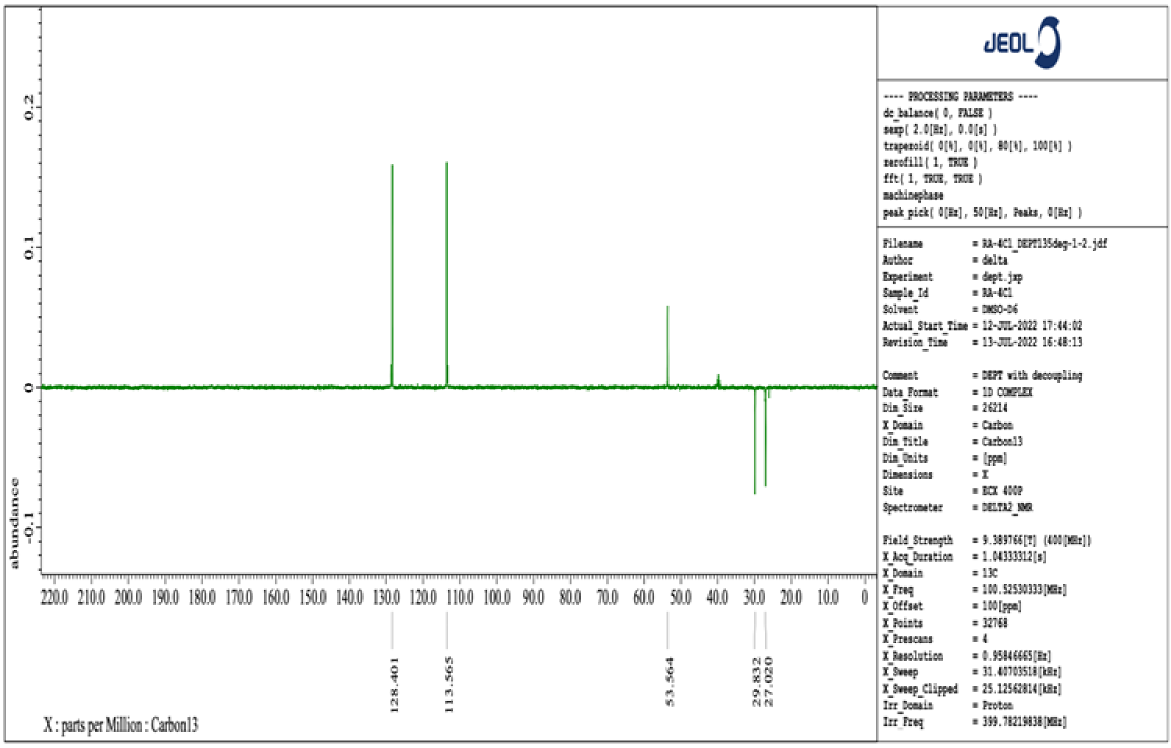

Figure S17: DEPT spectrum of **RA-4Cl**

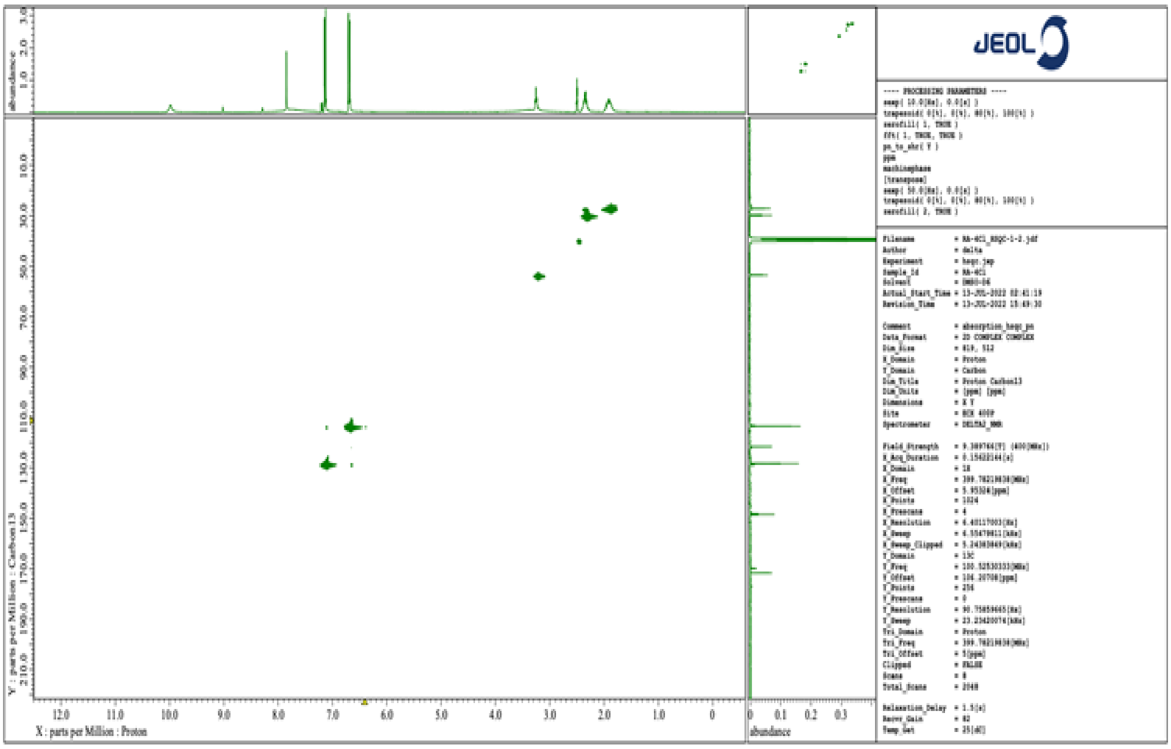

Figure S18: HMQC spectrum of **RA-4Cl**

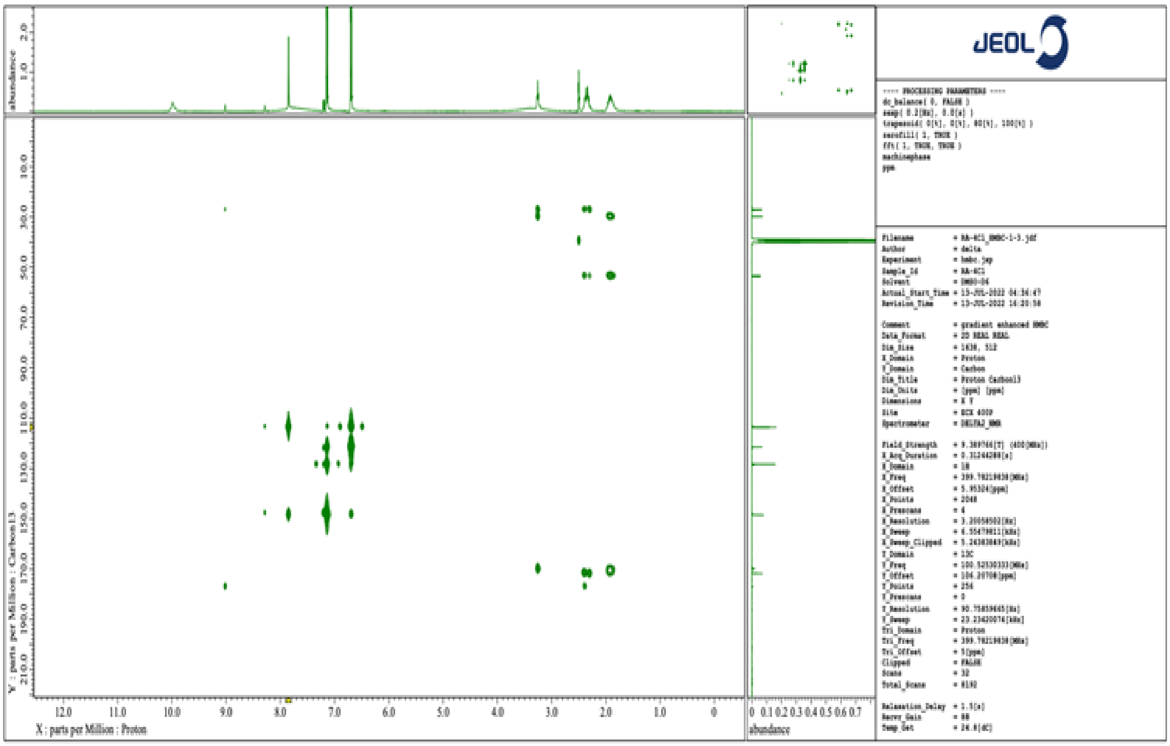

Figure S19: HMBC spectrum of **RA-4Cl**

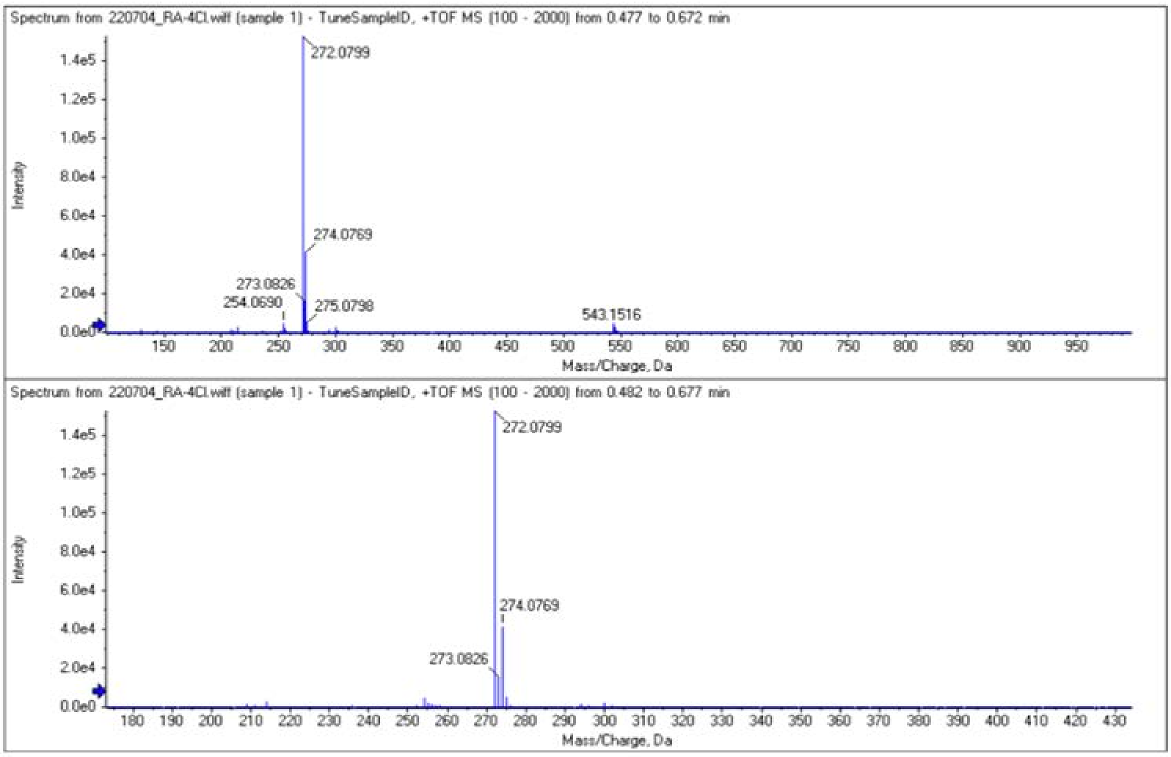

Figure S20: HRESIMS spectrum of **RA-4Cl**

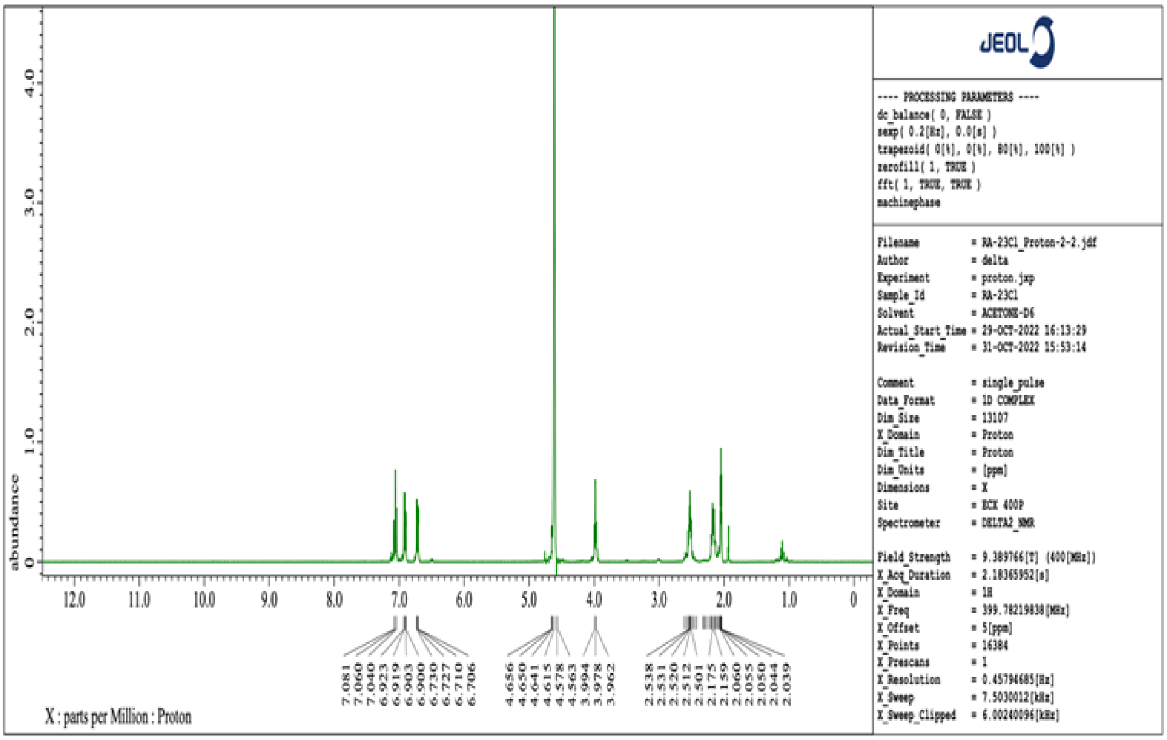

Figure S21: ^{1}H NMR (400 MHz) spectrum of **RA-23Cl**

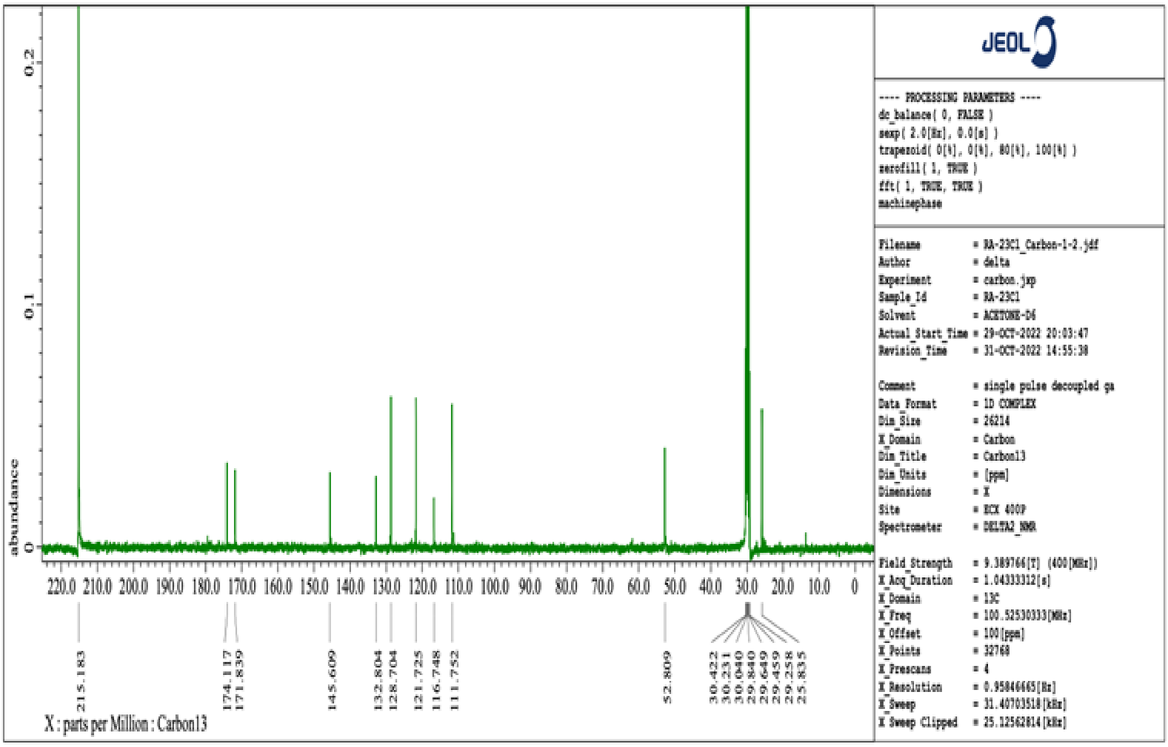

Figure S22: ^{13}C NMR (100 MHz) spectrum of **RA-23Cl**

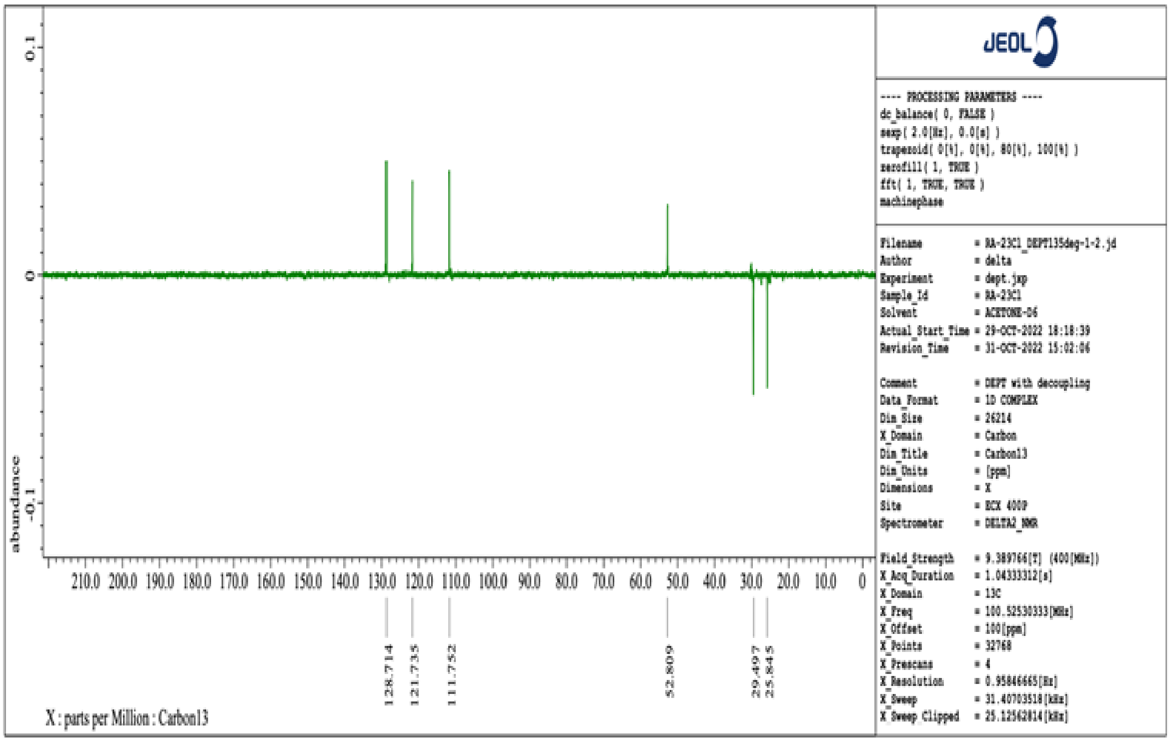

Figure S23: DEPT spectrum of **RA-23Cl**

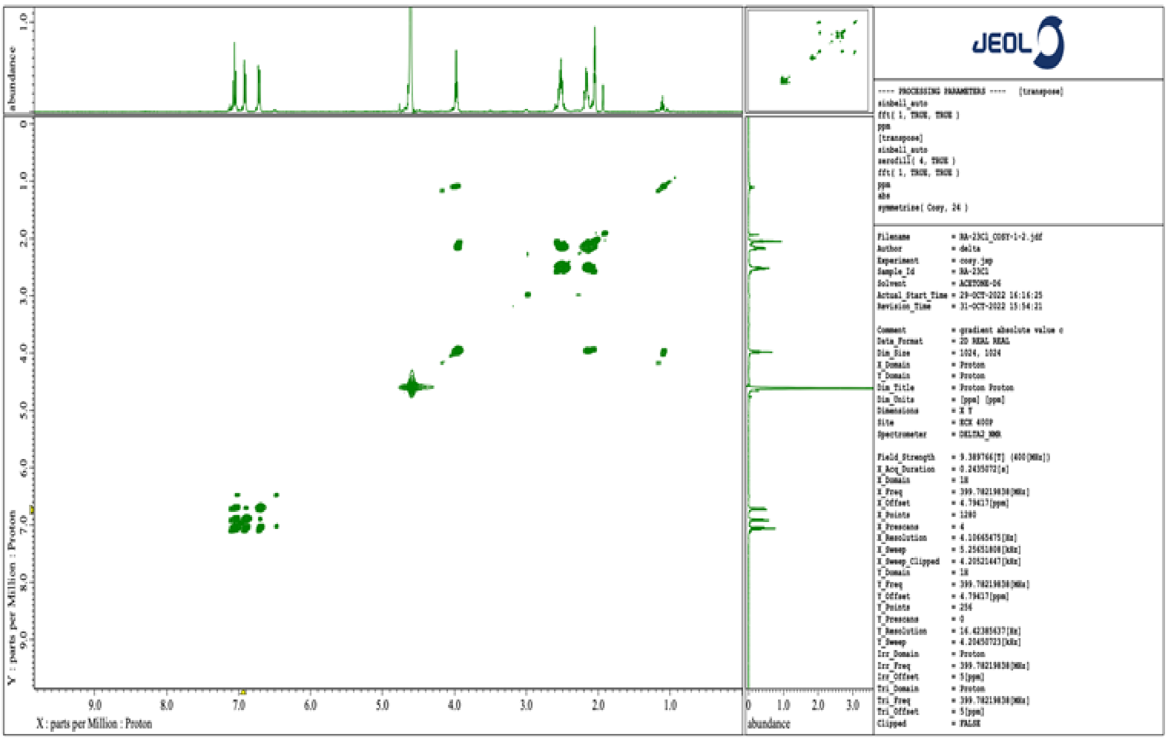

Figure S24: COSY spectrum of **RA-23Cl**

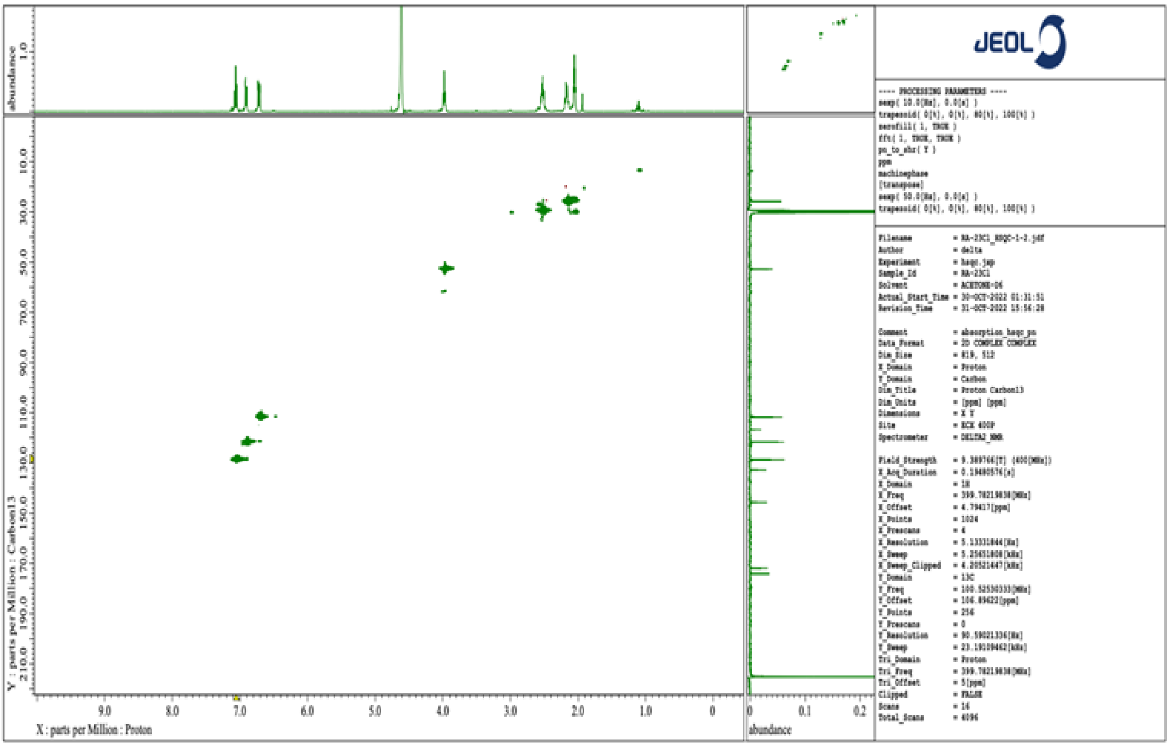

Figure S25: HMQC spectrum of **RA-23Cl**

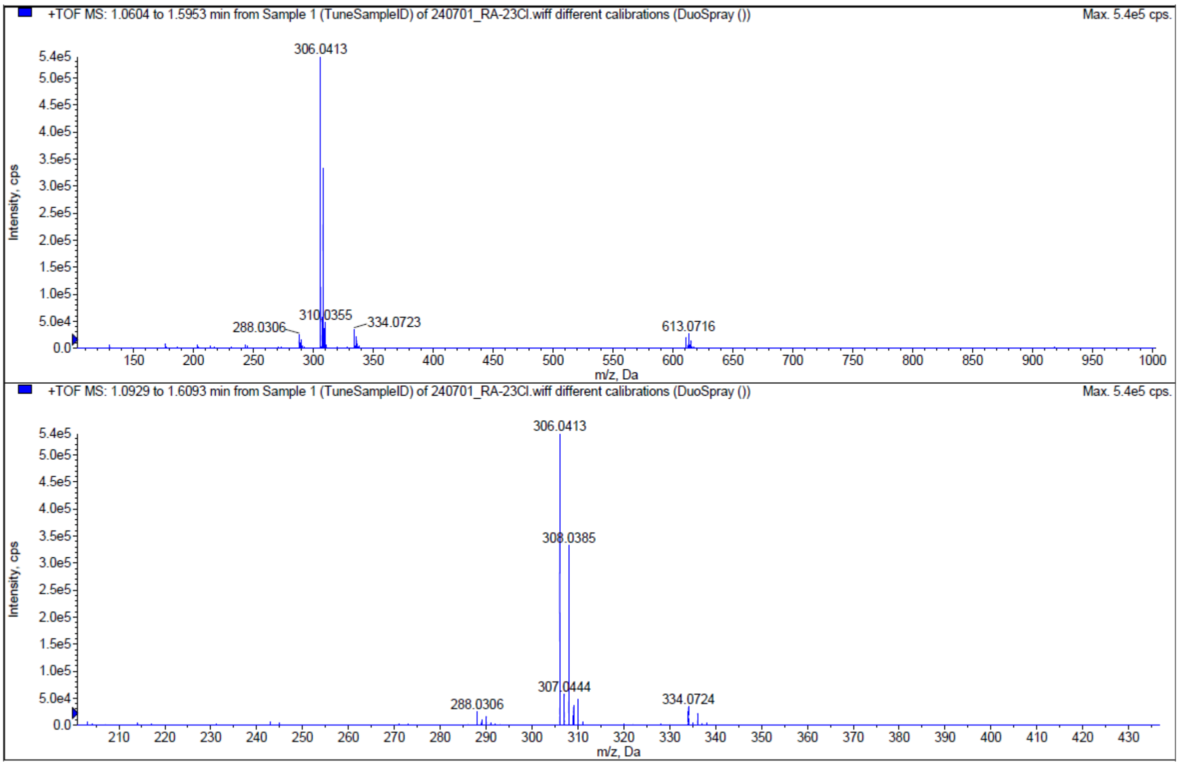

Figure S26: HRESIMS spectrum of **RA-23Cl**

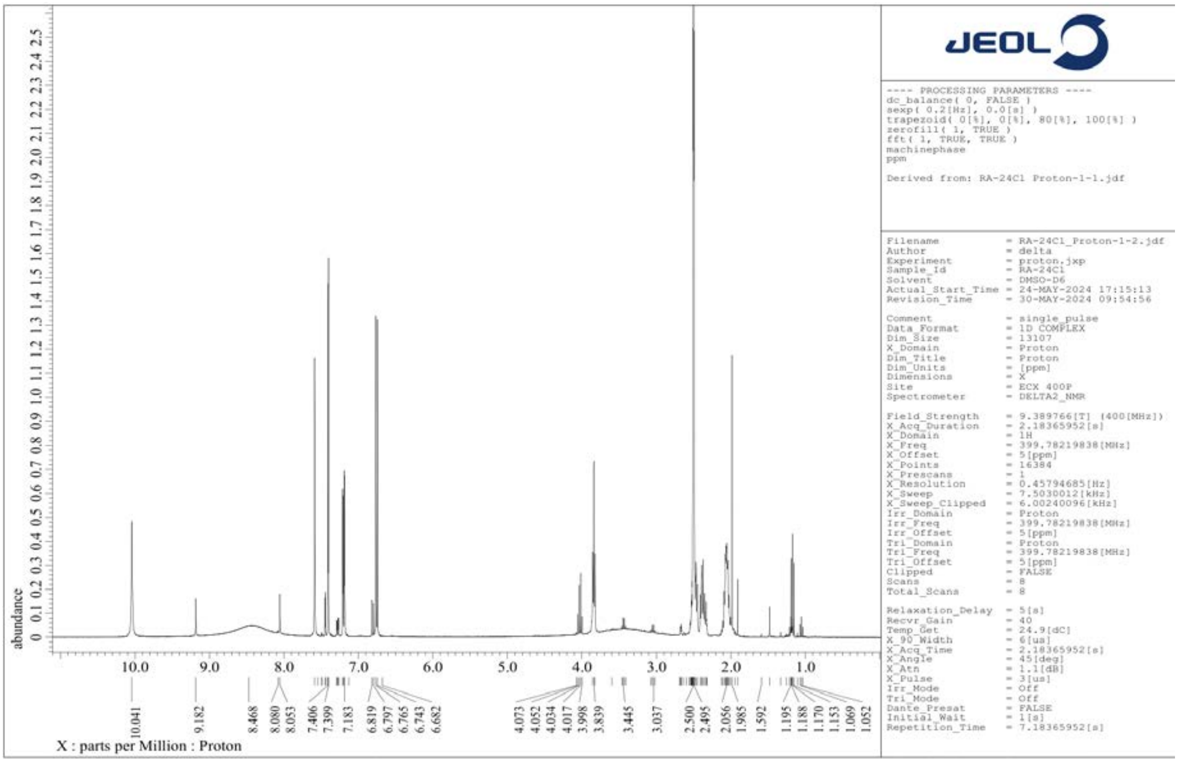

Figure S27: ^{1}H NMR (400 MHz) spectrum of **RA-24Cl**

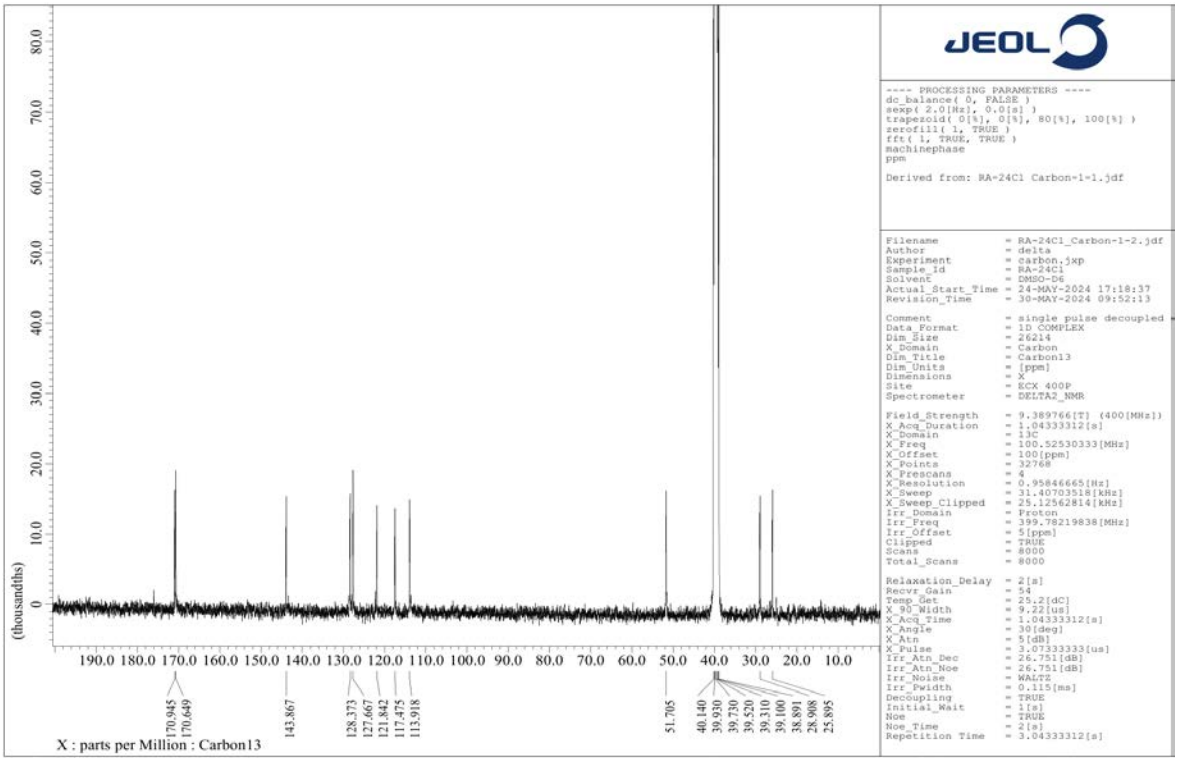

Figure S28: ^{13}C NMR (100 MHz) spectrum of **RA-24Cl**

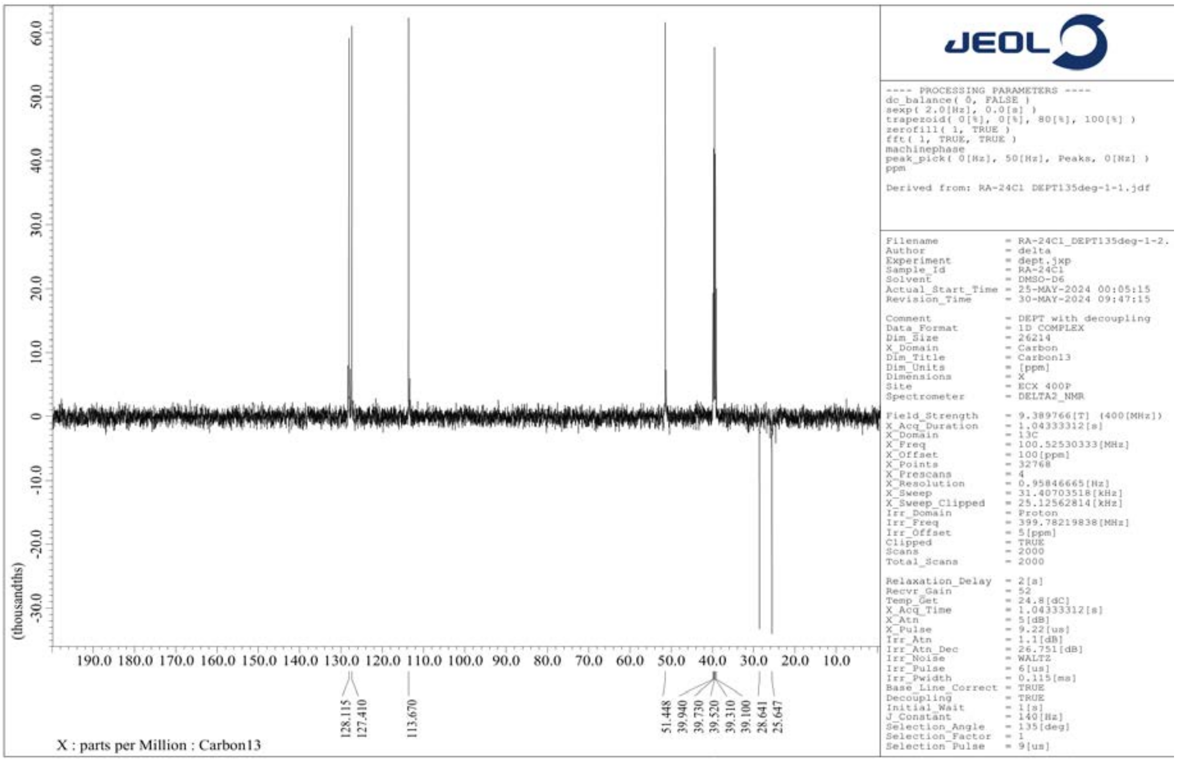

Figure S29: DEPT spectrum of **RA-24Cl**

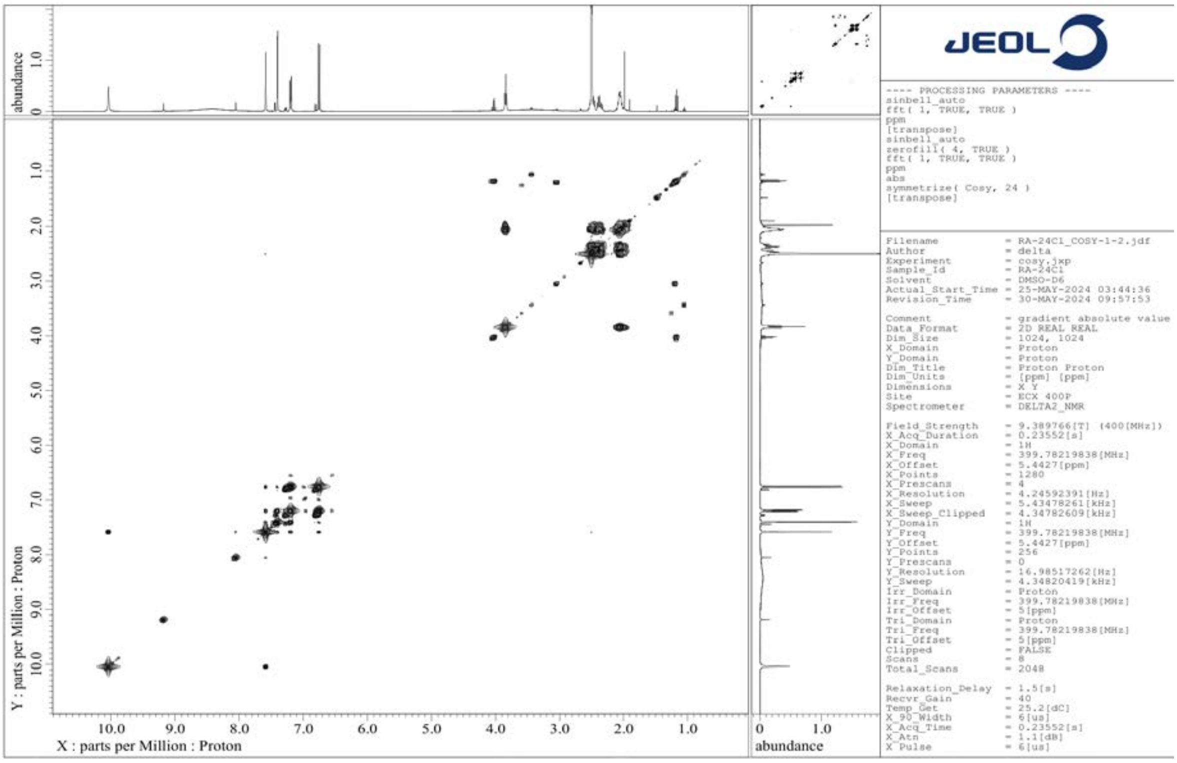

Figure S30: COSY spectrum of **RA-24Cl**

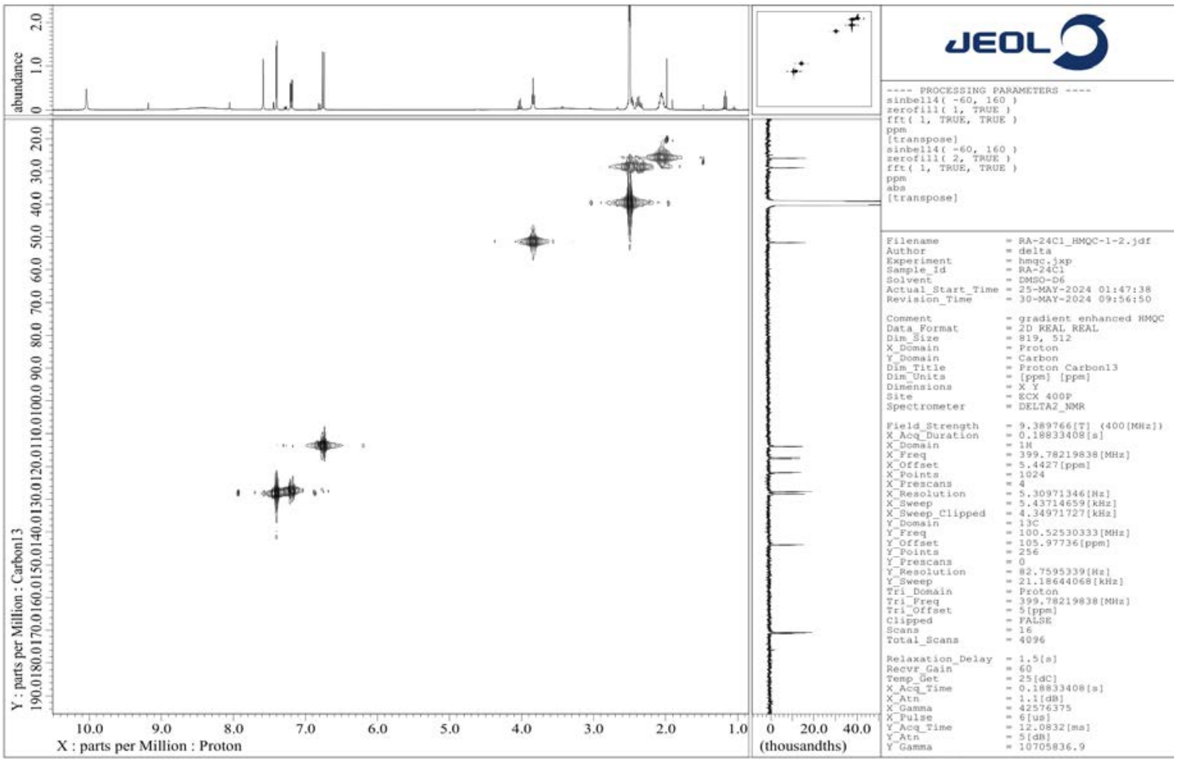

Figure S31: HMQC spectrum of **RA-24Cl**

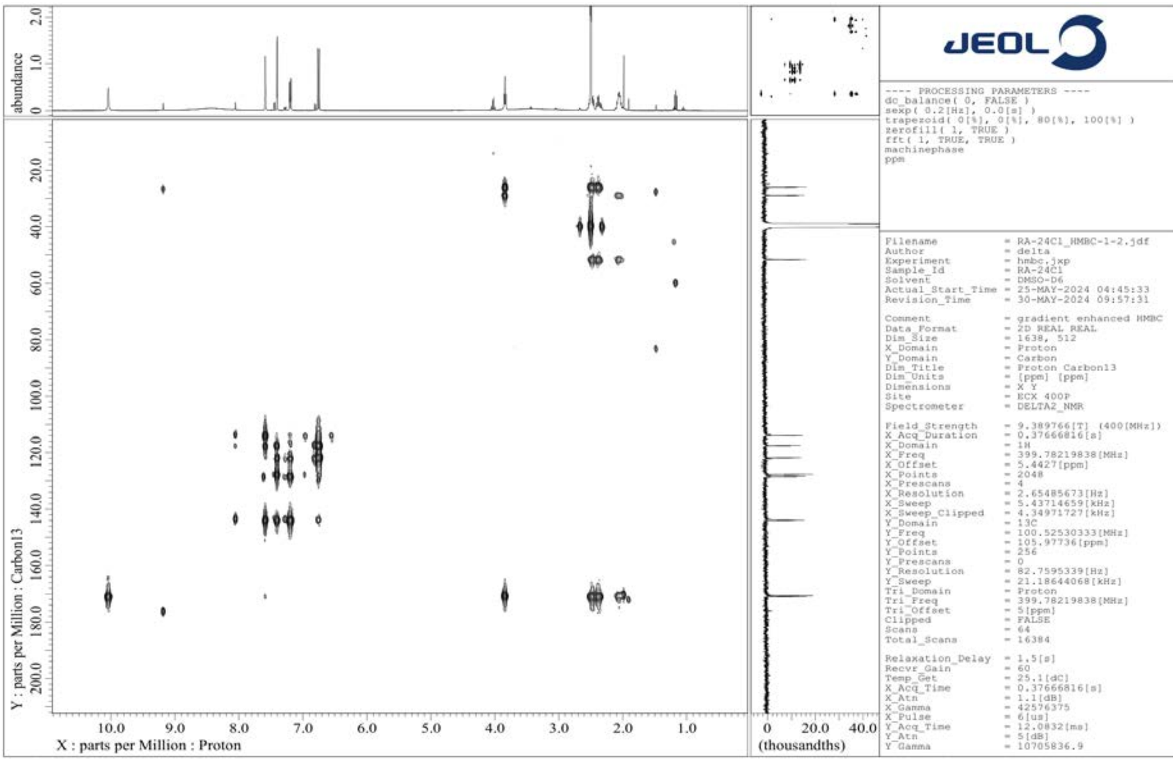

Figure S32: HMBC spectrum of **RA-24Cl**

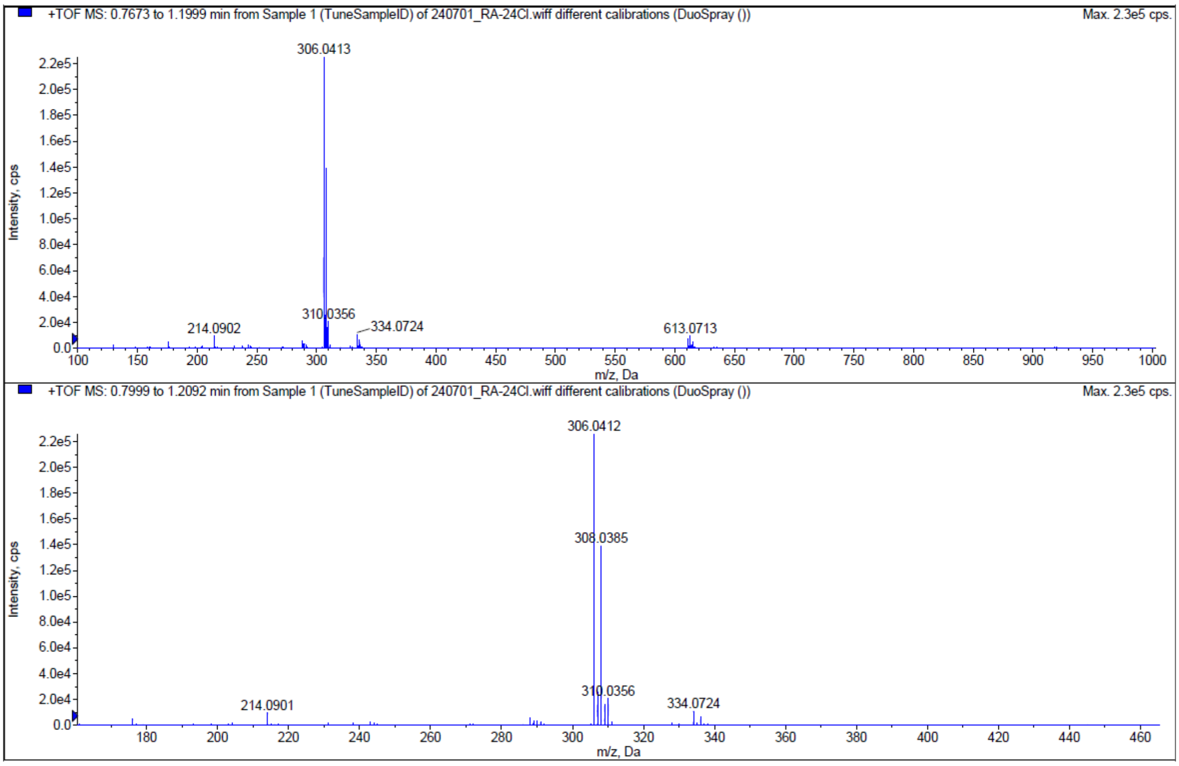

Figure S33: HRESIMS spectrum of **RA-24Cl**

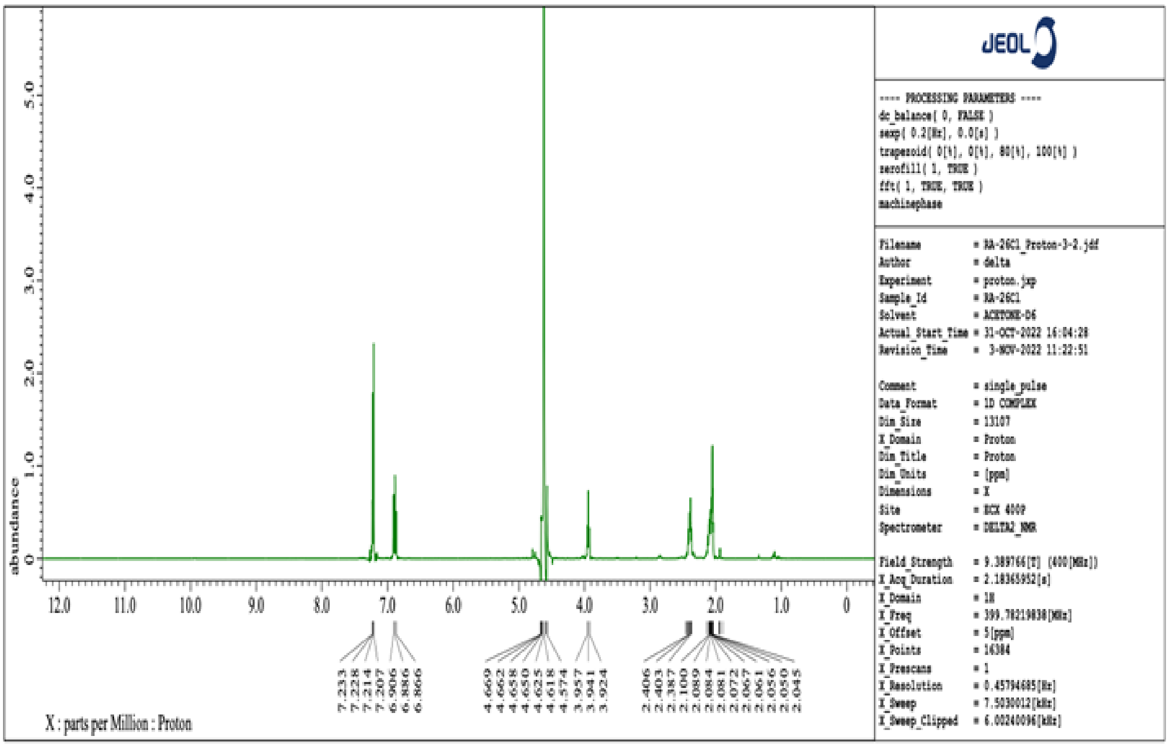

Figure S34: ^{1}H NMR (400 MHz) spectrum of **RA-26Cl**

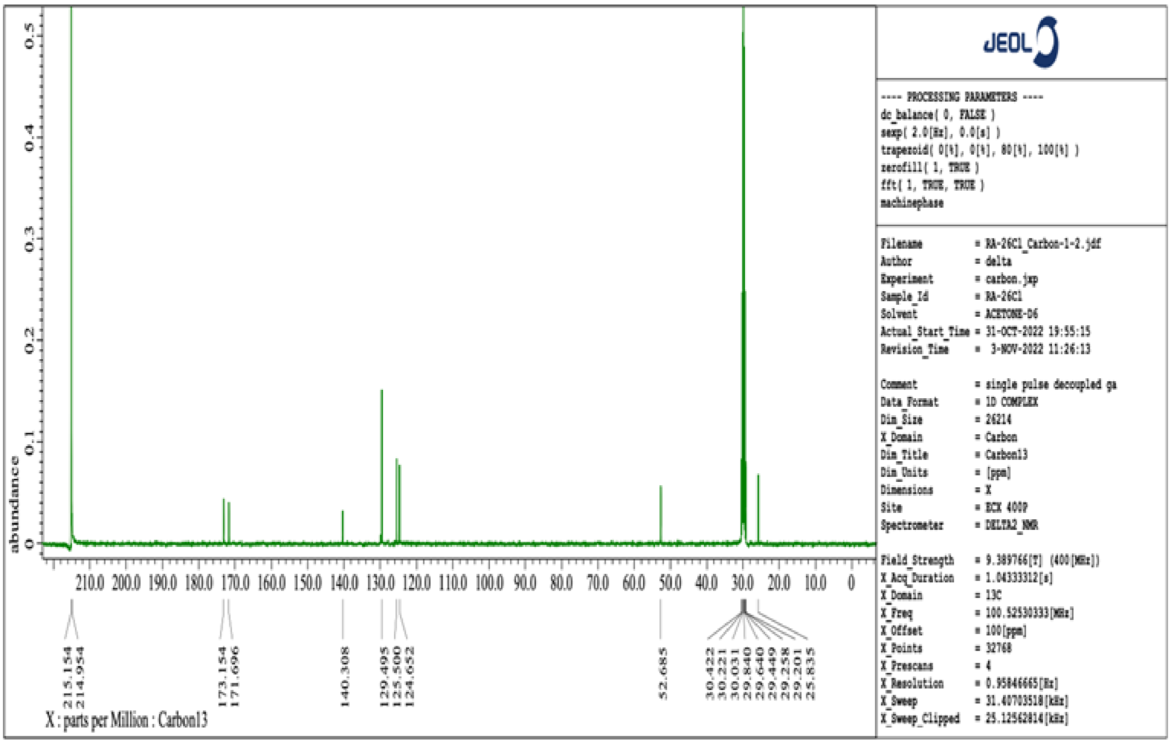

Figure S35: ^{13}C NMR (100 MHz) spectrum of **RA-26Cl**

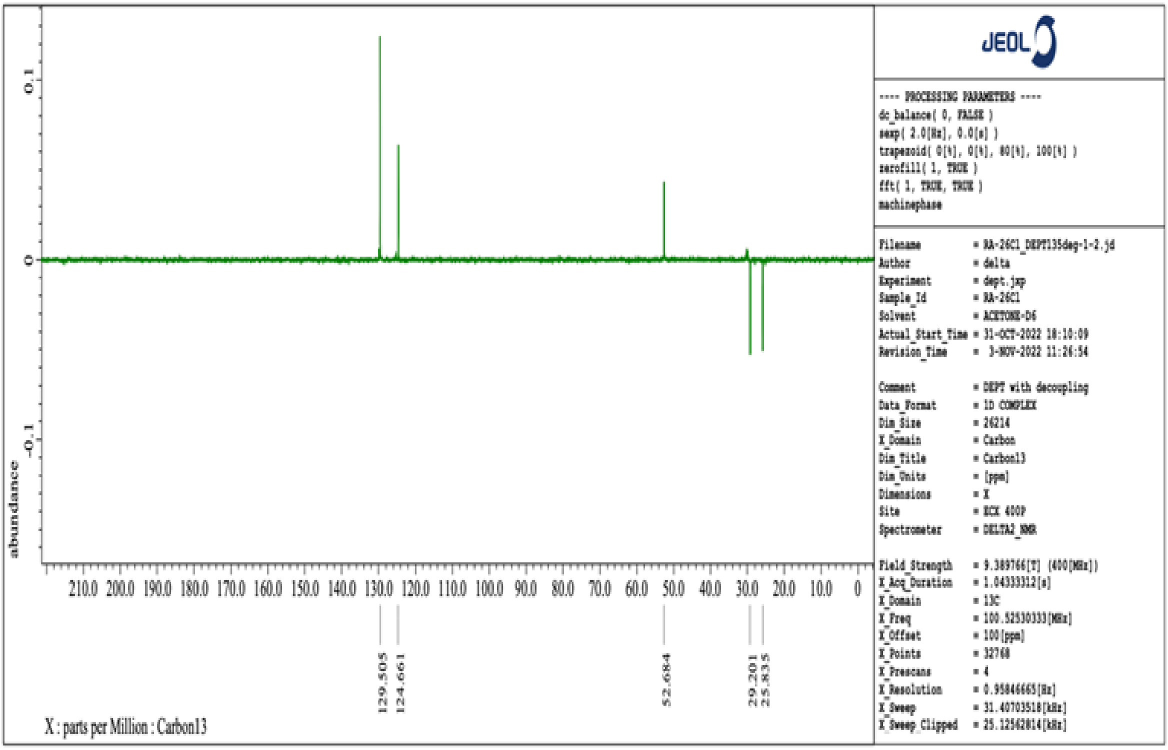

Figure S36: DEPT spectrum of **RA-26Cl**

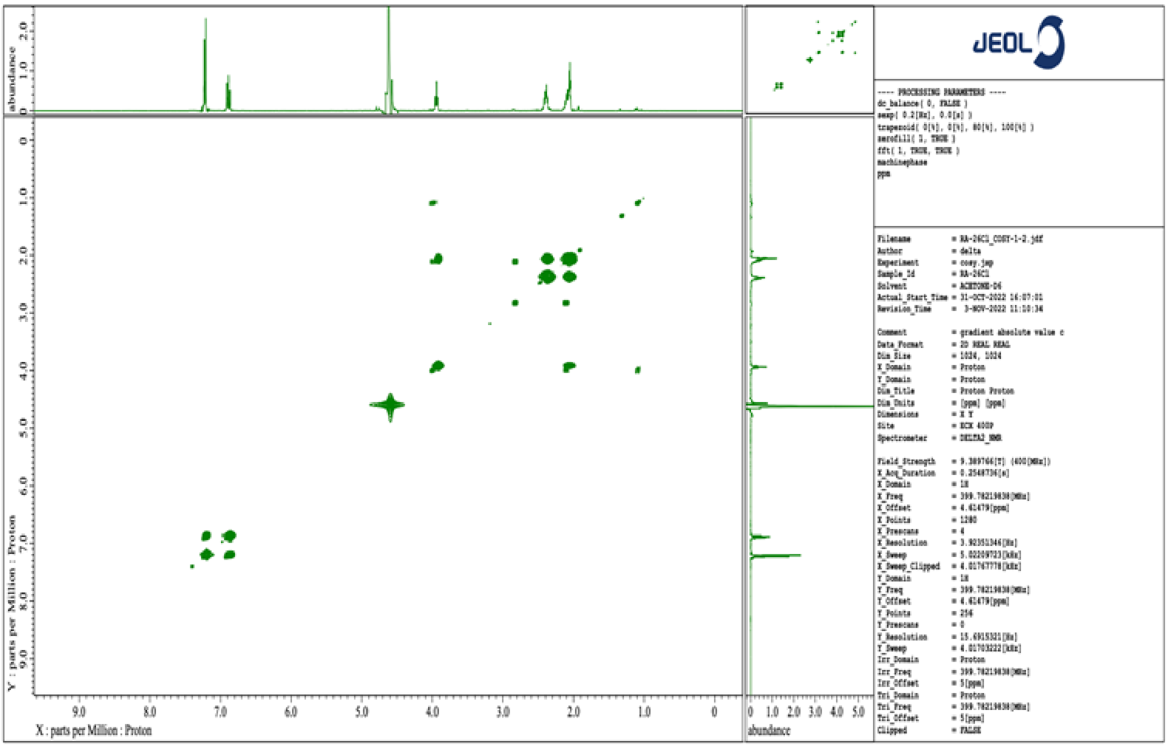

Figure S37: COSY spectrum of **RA-26Cl**

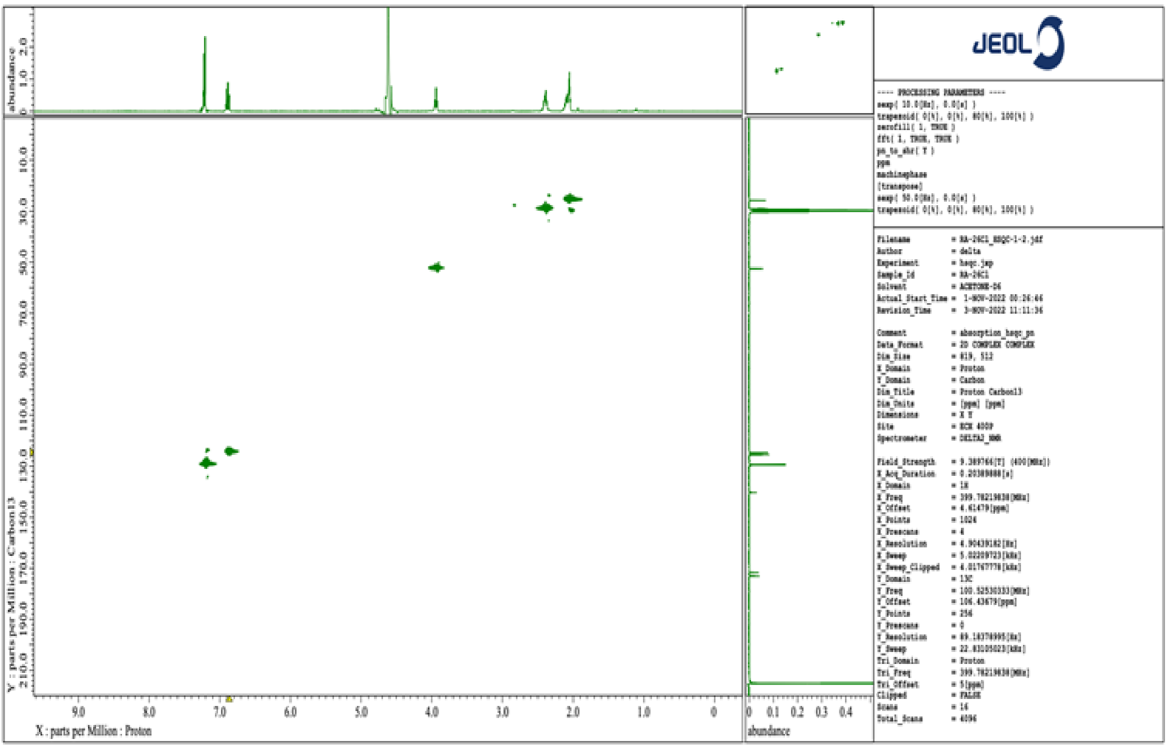

Figure S38: HMQC spectrum of **RA-26Cl**

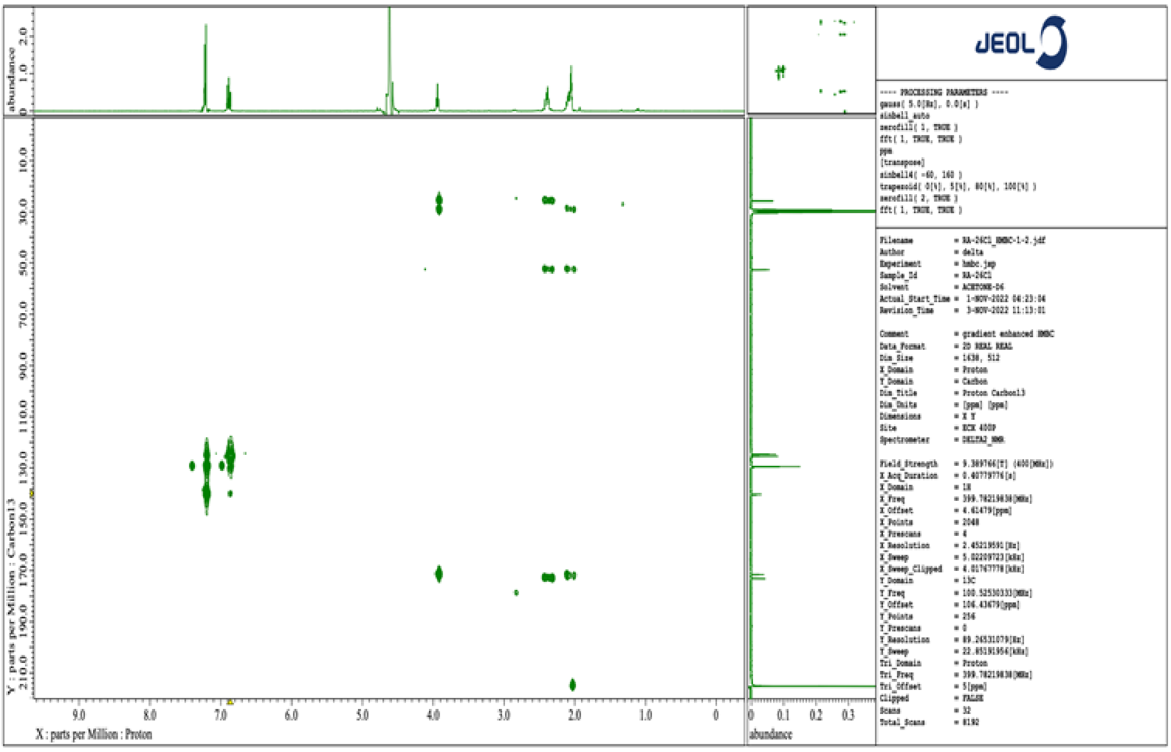

Figure S39: HMBC spectrum of **RA-26Cl**

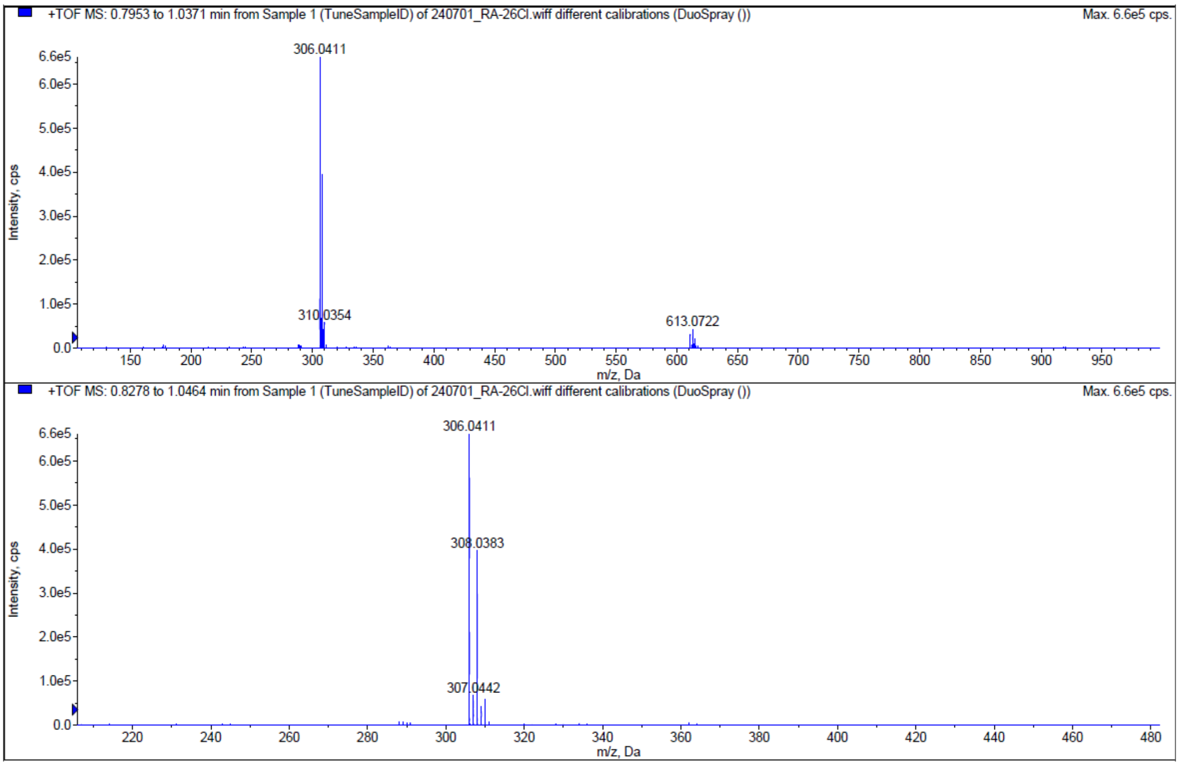

Figure S40: HRESIMS spectrum of **RA-26Cl**

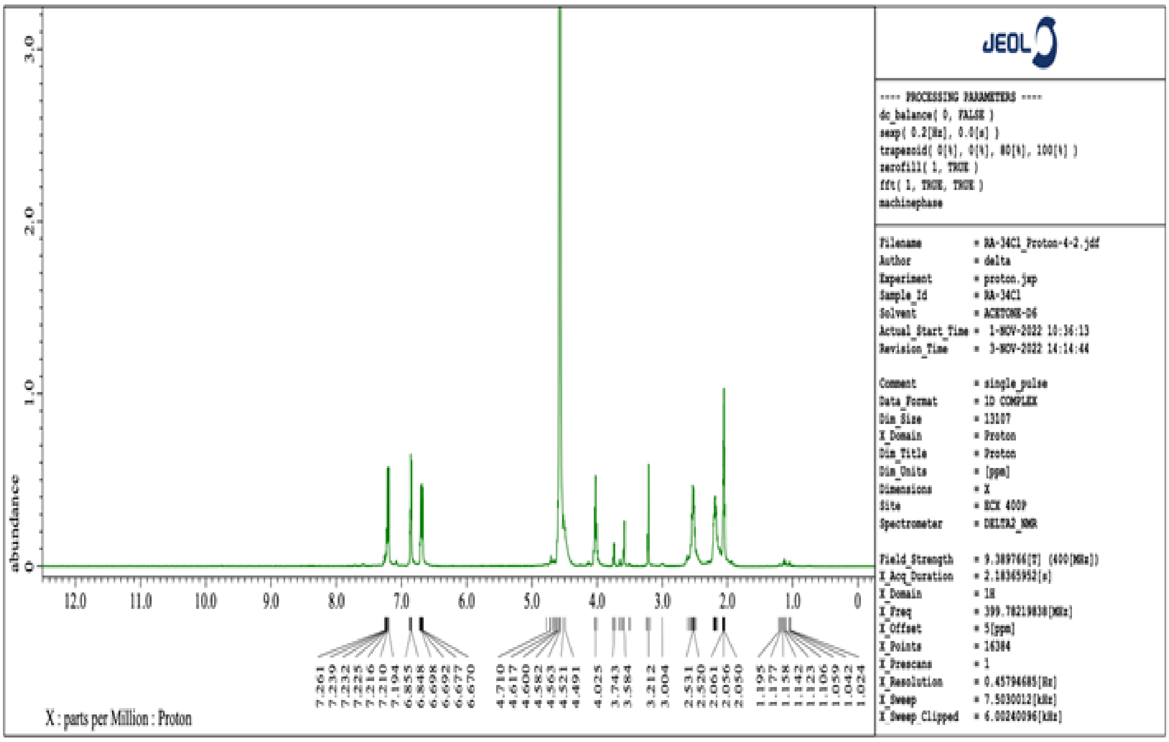

Figure S41: ^{1}H NMR (400 MHz) spectrum of **RA-34Cl**

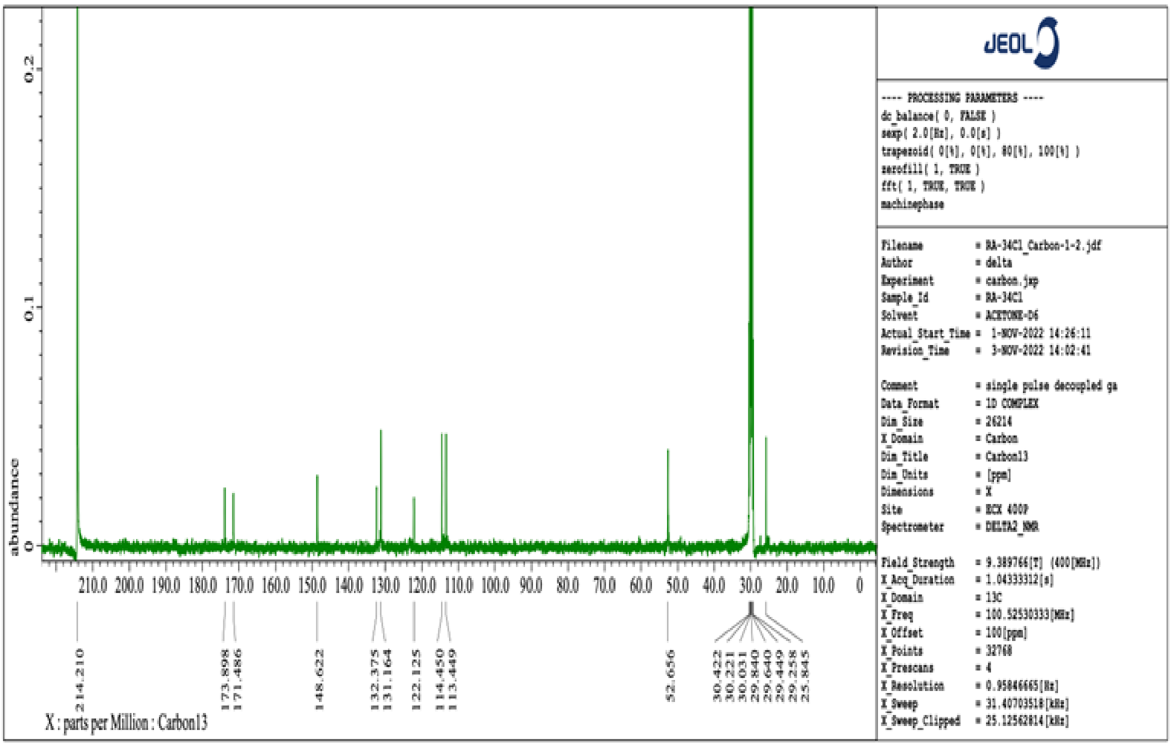

Figure S42: ^{13}C NMR (100 MHz) spectrum of **RA-34Cl**

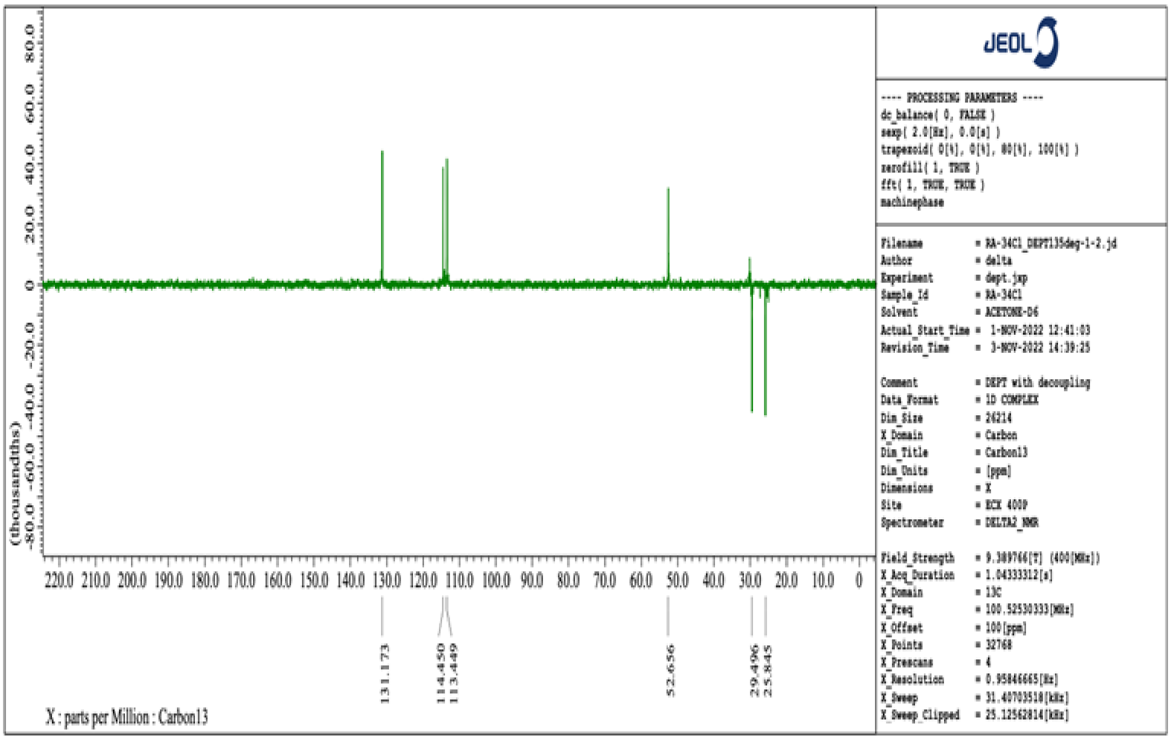

Figure S43: DEPT spectrum of **RA-34Cl**

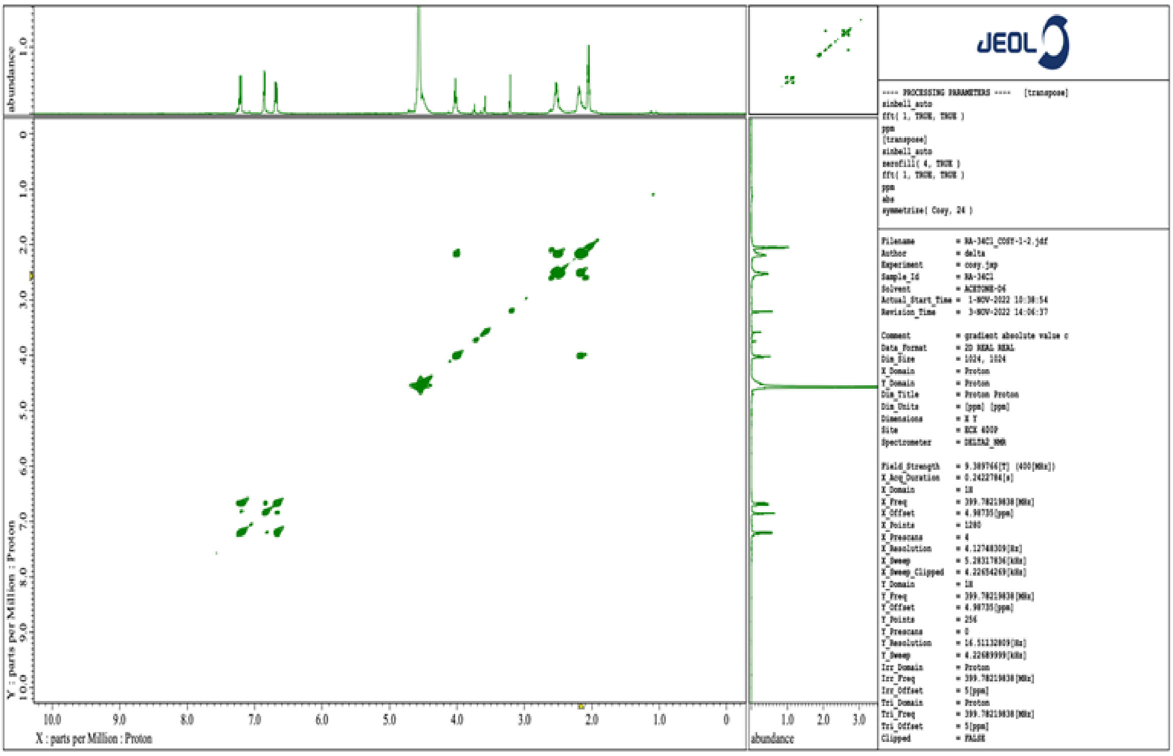

Figure S44: COSY spectrum of **RA-34Cl**

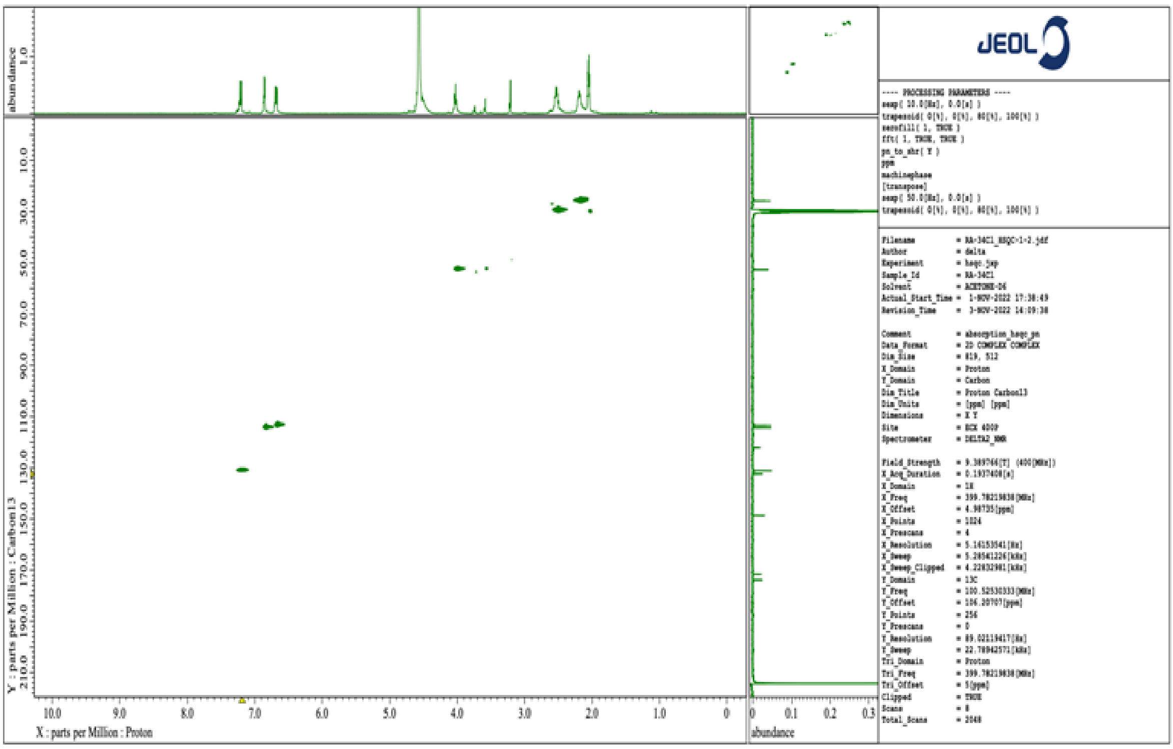

Figure S45: HMQC spectrum of **RA-34Cl**

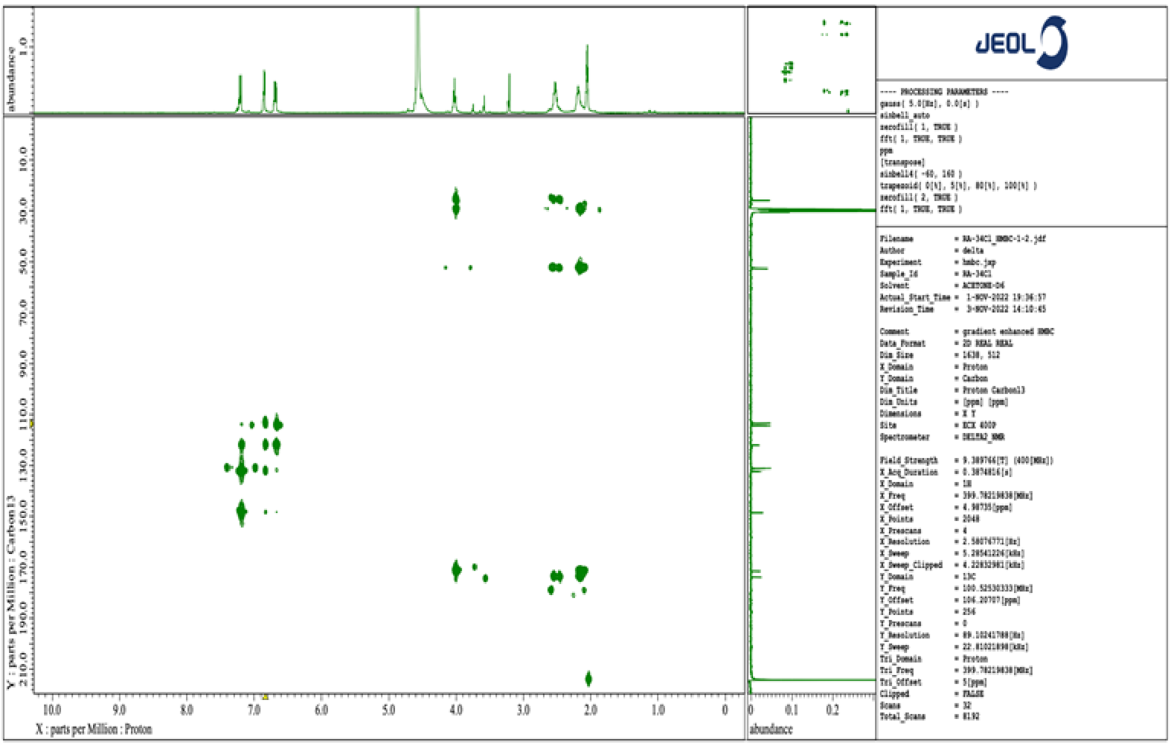

Figure S46: HMBC spectrum of **RA-34Cl**

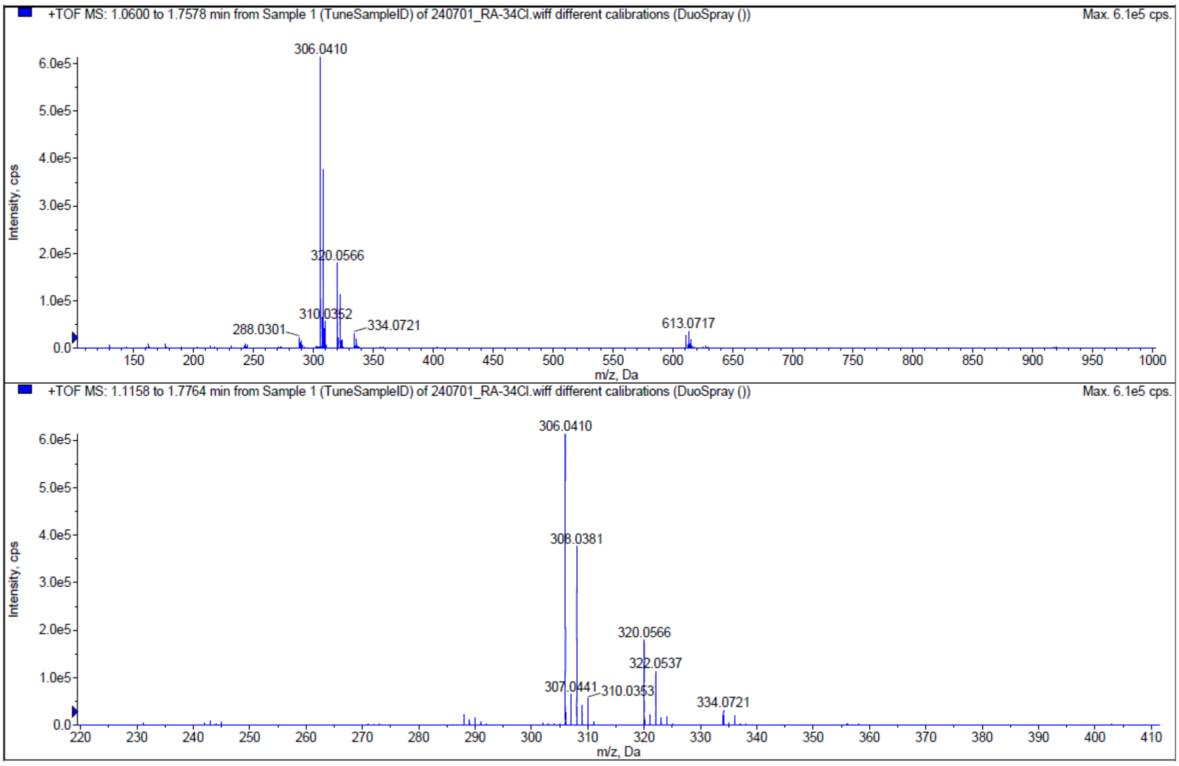

Figure S47: HRESIMS spectrum of **RA-34Cl**

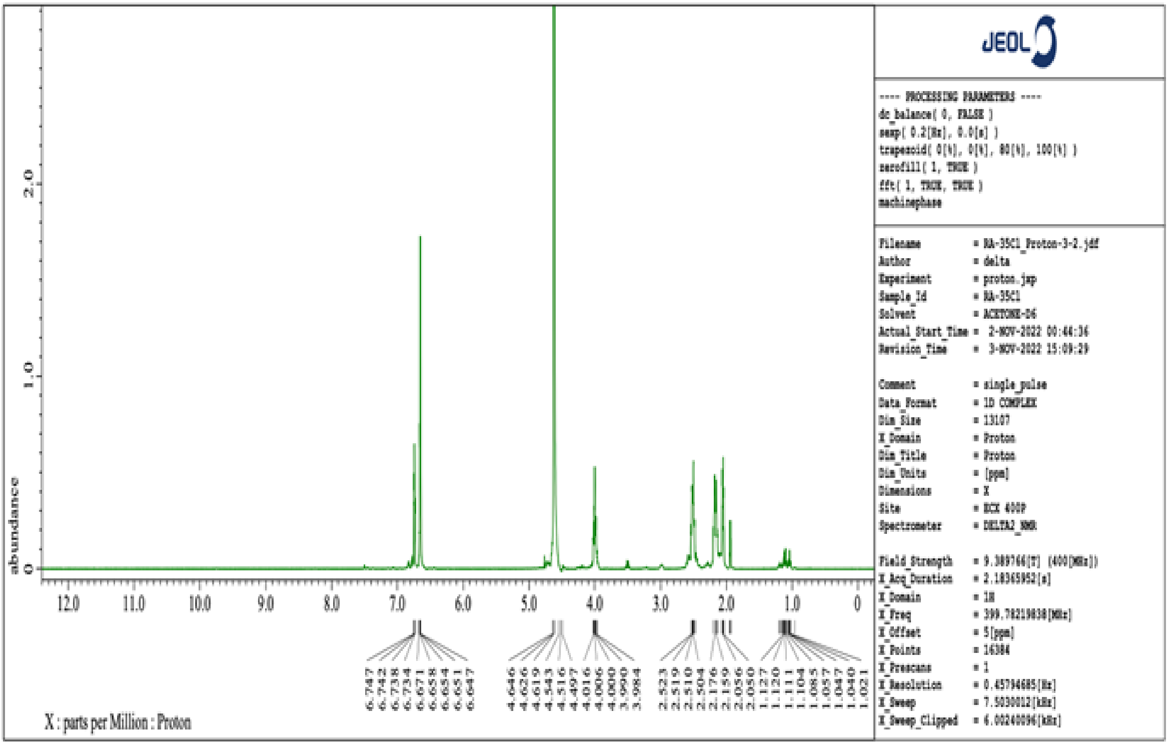

Figure S48: ^{1}H NMR (400 MHz) spectrum of **RA-35Cl**

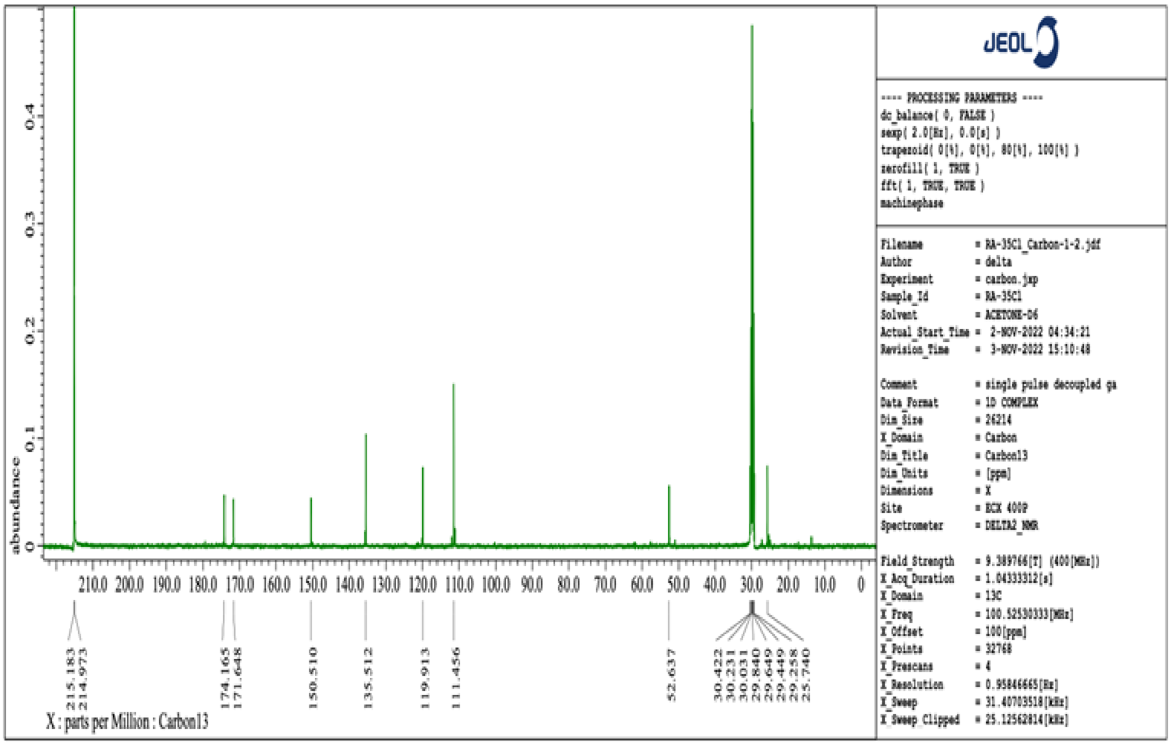

Figure S49: ^{13}C NMR (100 MHz) spectrum of **RA-35Cl**

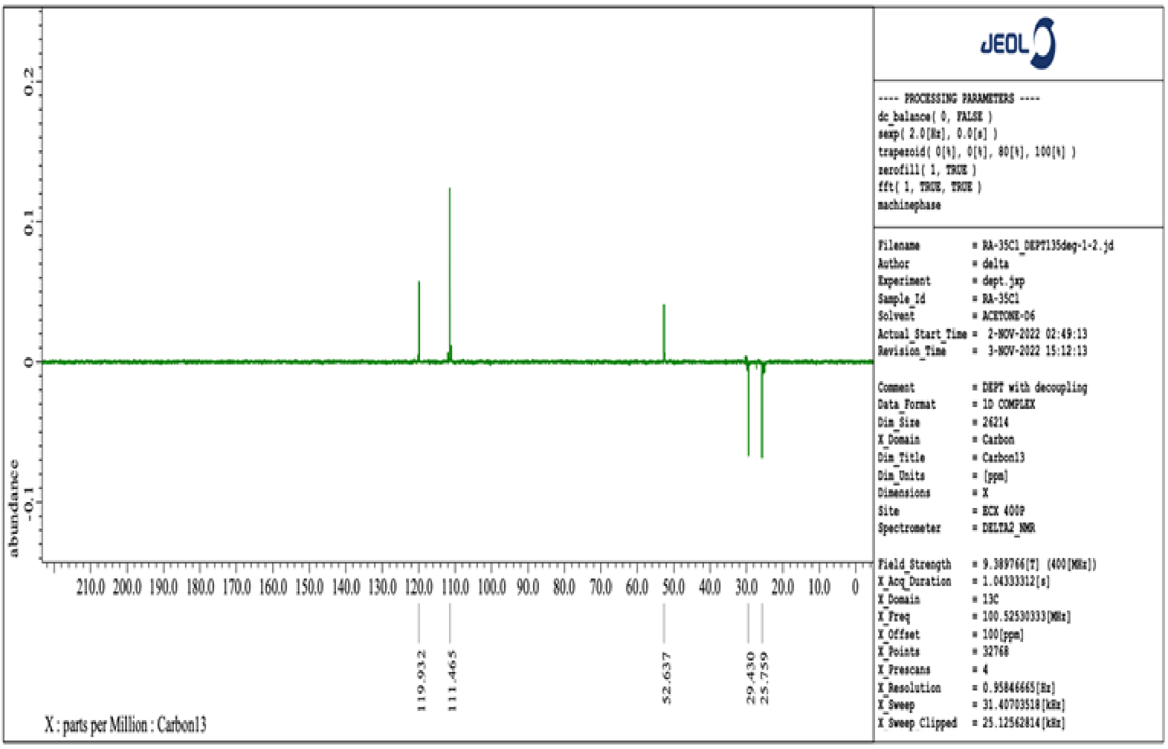

Figure S50: DEPT spectrum of **RA-35Cl**

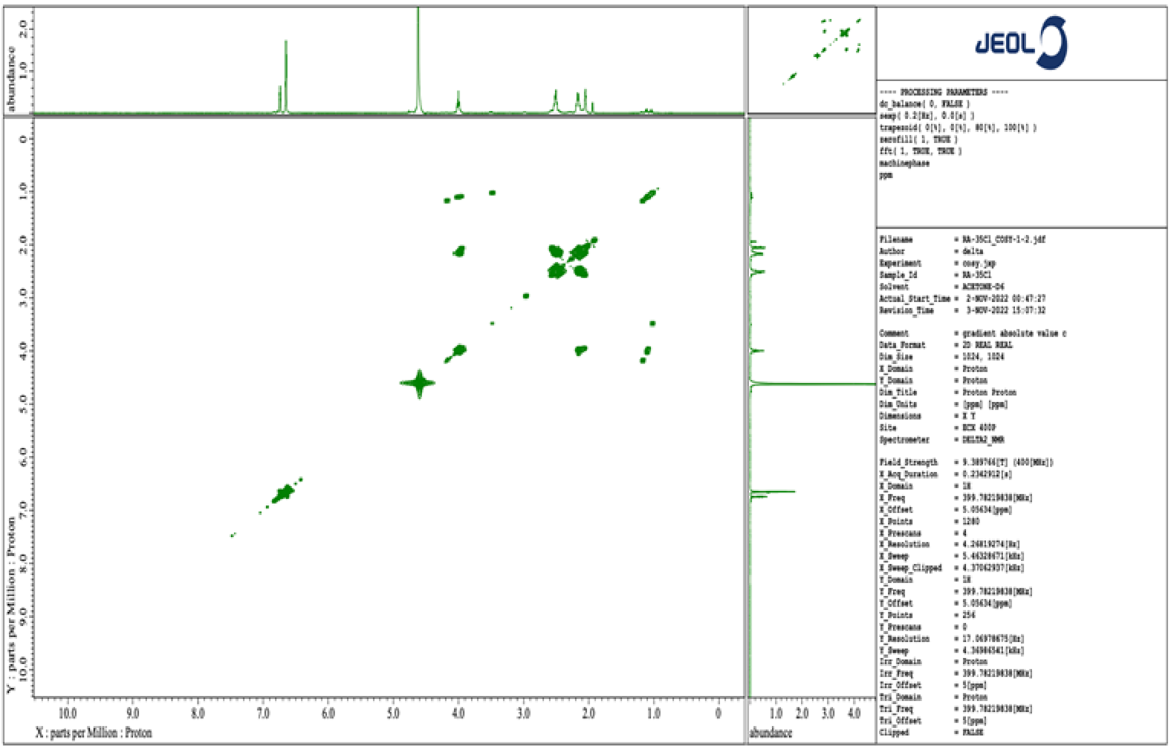

Figure S51: COSY spectrum of **RA-35Cl**

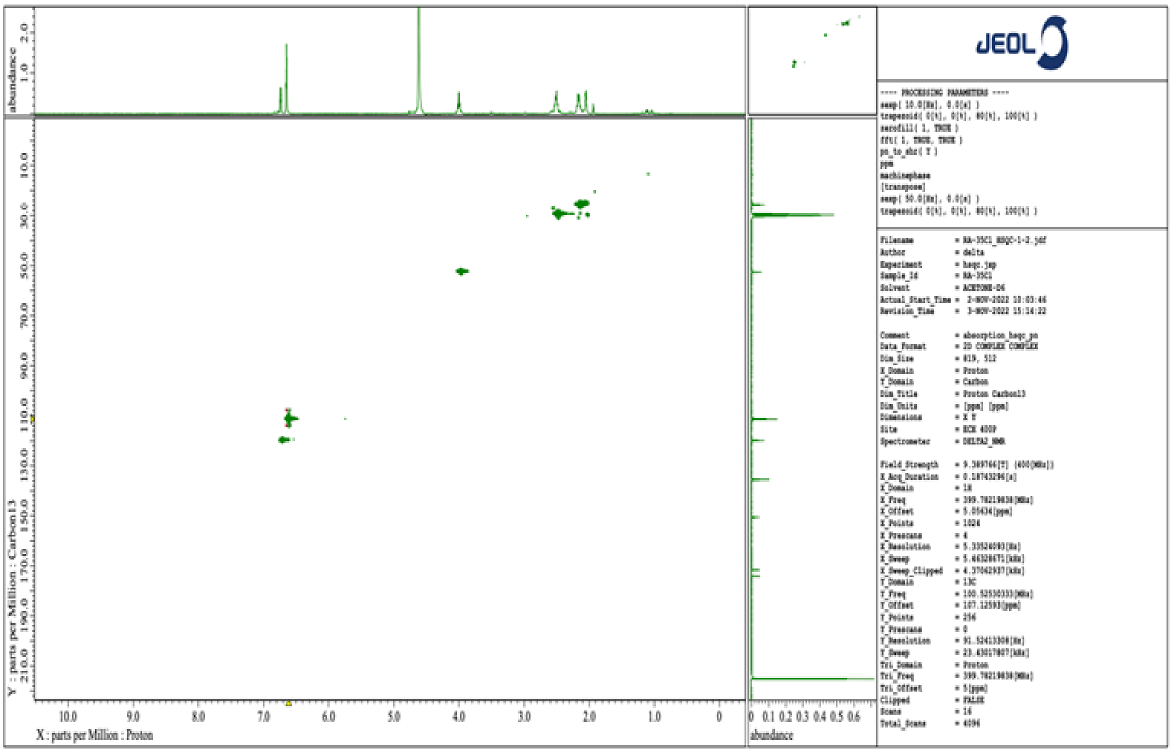

Figure S52: HMQC spectrum of **RA-35Cl**

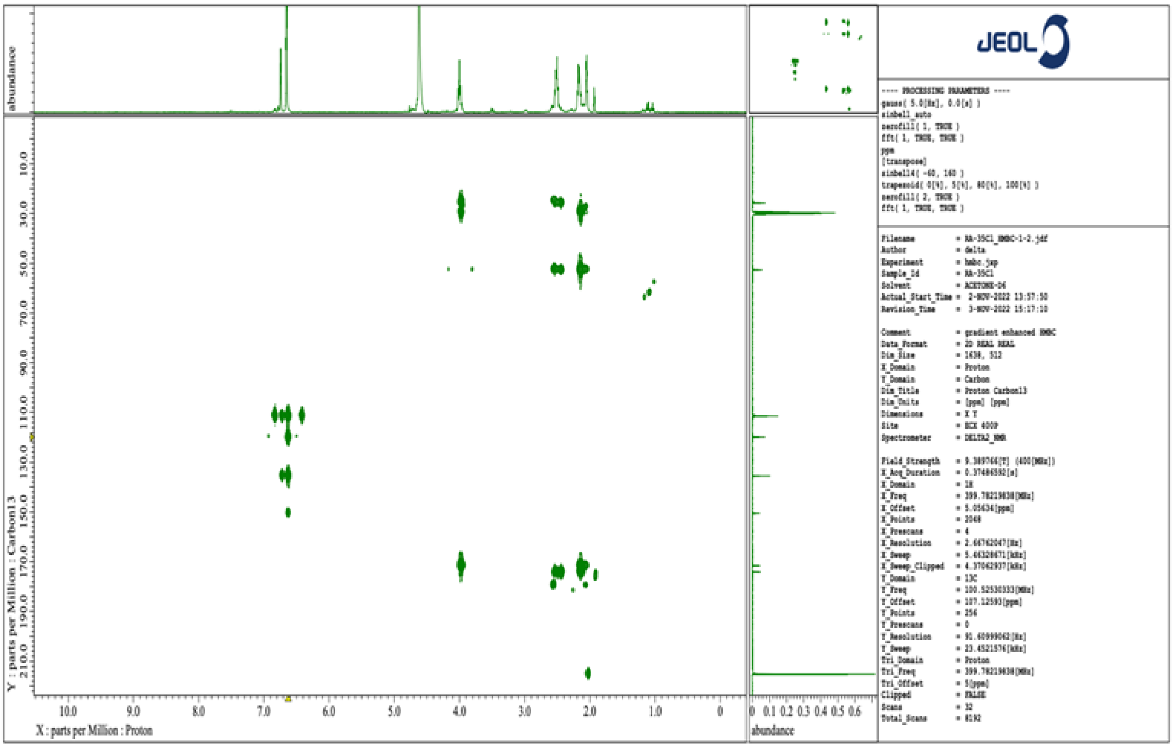

Figure S53: HMBC spectrum of **RA-35Cl**

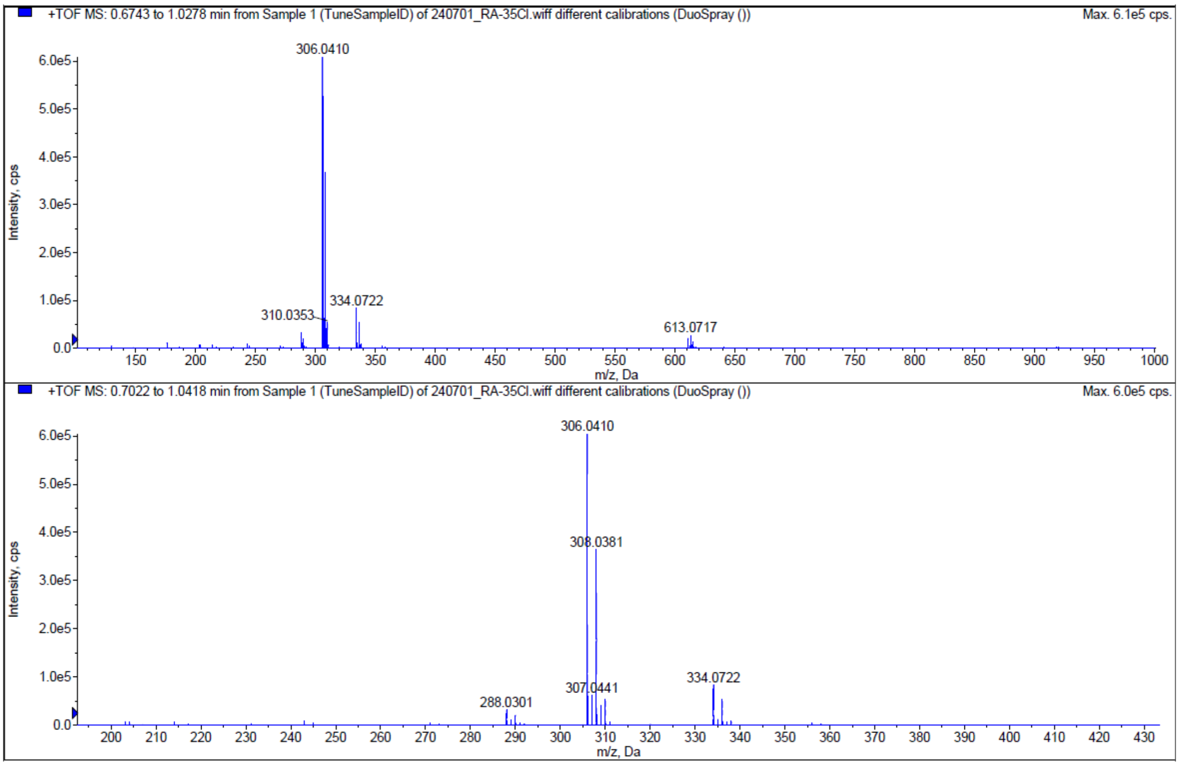

Figure S54: HRESIMS spectrum of **RA-35Cl**
